# Whole-Genome Analysis of LSDV Isolates from the 2019 and 2023 Outbreaks in Israel Points to Undetected Circulation and Recombination Events

**DOI:** 10.3390/vetsci13040333

**Published:** 2026-03-30

**Authors:** Praveen Kumar Verma, Manoj Kumar, Marisol Rubinstein-Guini, Sharon Karniely, Elad Eliahoo

**Affiliations:** Department of Virology, Kimron Veterinary Institute, Ministry of Agriculture and Food Security, Beit Dagan 5025001, Israel; praveen.verma@mail.huji.ac.il (P.K.V.);

**Keywords:** LSDV, WGS, whole-genome comparison, codon usage, recombination events

## Abstract

Lumpy skin disease virus (LSDV) is a large DNA capripoxvirus that causes LSD, a disease that has major economic impact. Since 1989, several sporadic outbreaks were reported in Israel, with the latest outbreaks in 2012, 2019 and 2023. Although considered genetically stable, LSDV shows a high degree of genetic recombination events and genetic variations. In particular, in-frame nonsense mutations were suggested to act as one of the main evolutionary drivers of outbreaks. Whole-genome sequencing of LSDV isolates from the 2019 and 2023 outbreaks was used for genomic analysis using various bioinformatics tools to characterize the genomic evolution, recombination events and micro-evolutionary forces shaping LSDV in Israel by comparing isolates. Comparative genomic analysis revealed substantial nucleotide substitutions in the 2019 and 2023 isolates relative to the 2012 isolate. Specifically, increased nucleotide mismatches, inter-genic deletion, enhanced APOBEC editing signatures and elevated codon usage. Additionally, numerous mutations were recognized, leading to structural disruptions in specific viral proteins and possible RNA instability. In conclusion, this analysis supports that nucleotide substitutions, codon selection pressure and APOBEC-associated editing had driven local microevolution of LSDV during the years between outbreaks despite the absence of clinical indications and major vaccination campaigns. Furthermore, genomic evidences of recombination events between the 2012 and 2019 isolates suggests that these processes may have contributed to the emergence of the variant identified during the 2023 outbreak.

## Simple Summary

Lumpy skin disease virus (LSDV) is a capripoxvirus causing lumpy skin disease (LSD), a skin disease in cows with distinct lesions. The disease was reported in Africa, the Middle East, Asia and south Europe and has major economic impact on countries during outbreaks. In Israel the disease was first reported in 1989, with subsequent outbreaks occurring in 2012, 2019 and 2023. In this work, whole-genome comparison was obtained on isolates from the 2019 and 2023 outbreaks in reference to the 2012 isolate. In both the 2019 and 2023 isolates, genomic differences including substantial nucleotide substitutions, increased nucleotide mismatches, inter-genic deletion, enhanced APOBEC editing signatures and elevated codon usage were observed. Additionally, numerous mutations were recognized, leading to structural disruptions in specific viral proteins and possible RNA instability. Therefore, despite the absence of clinical indications and major vaccination campaigns, genomic evidence supports the circulation of the 2012 and 2019 variants during the years between the outbreaks with recombination events between them that may have contributed to the emergence of the variant identified during the 2023 outbreak.

## 1. Introduction

Lumpy skin disease virus (LSDV), the etiological agent of lumpy skin disease (LSD), is a double-stranded DNA virus which belongs to genus *Capripoxvirus* of the family *Poxviridae* [1]. The LSDV genome is 145 to 152 kbp in size, with a central coding region bounded by two inverted terminal repeat regions. Although LSDV maintains genome stability, it is known to have a high degree of genetic recombination between strains [2]. LSDV is endemic in Africa, but in the last several decades, outbreaks have been reported in the Middle East, south Europe, Russia and sub-continental Asia. Moreover, recent incursions of LSDV were reported in Indonesia in 2022; Japan in 2024; and Italy, France, and Spain in mid-2025 [3,4]. Since its first discovery in Zambia in 1929, it has differentiated into two major phylogenetic clusters (1.1 and 1.2), with multiple sub-strains, including the historic Neethling prototype strain, and five unique recombinant strains (clusters 2.1–2.5) [5]. This viral disease has major impact on the economies of affected countries due to decreased dairy production, restrictions on export of meat/beef and the direct economic damage to farmers [6]. The disease is mainly transmitted mechanically through blood-sucking arthropods and affects bovine inhabitants, which show a plethora of specific clinical signs varying from subclinical infection to death [7]. The main clinical signs of infected bovines are nodule development, fever, lesions on the mouth, skin edema and lymph node enlargement. In addition, it was seen that after infection, cows lose weight, produce less milk, and might develop severe health issues that can lead to death [8].

LSDV outbreaks were first reported in Israel in 1989, and subsequent outbreaks in Israel were recorded in 2006 and in 2012–2013 [9]. Following the major outbreak of 2012–2013 in Israel, the veterinary services instituted three years of mandatory vaccination against the virus. Despite this, two additional outbreaks were detected in Israel—in the summer of 2019 and again in the summer of 2023—affecting several cattle and dairy farms. The 2019 outbreak had been identified in 17 sites, mainly in the north of the country, and originated from the north-east (Syrian border) (Israel Veterinary Services report, 2019, https://www.gov.il/BlobFolder/guide/dohot-shnatim-vet/he/vet_doch_shnati_2019.pdf, accessed on 26 March 2026). The 2023 outbreak had been identified on three farms in the south part of Israel and originated from the south-west (Egyptian border) (Israel Veterinary Services report, 2023, https://www.gov.il/BlobFolder/guide/dohot-shnatim-vet/he/services_and_livestock_health_2023.pdf, accessed on 26 March 2026). The different origin of each virus, the 2019 isolate from Asia and the 2023 isolate from Africa, presents the potential risk of recombination of distinct strains to form a novel strain that might challenge the current treatments, prevention and vaccination protocols.

Therefore, it is important to apply genomic analysis on LSDV strains that have emerged in the same geographic region. For this purpose, we used whole-genome next-generation sequencing (WGS) supported by bioinformatics tools that were developed for genomic studies such as phylogenetic analysis, genetic distance, SNP analysis, codon usage and recombination analysis [10]. In particular, information regarding recombination events and the unique characteristic of each strain, that might influence the nature of the outbreaks, can be achieved by measuring the usage of synonymous codons with distinct frequencies in precise genes.

Codon usage bias is a universal phenomenon where usage of synonymous codons with different frequencies takes place, in which codon usage bias changes via natural selection, mutations or genetic drift in several organisms such as prokaryotes, eukaryotes and viruses. In particular, GC content, nucleotide composition, length of genes, mRNA folding and tRNA abundance are the few factors affecting codon bias and, thus, are participating in the evolution of the genetic code [11].

Analyzing these factors using whole-genome sequences of the LSDV variants that emerged in Israel in the last two outbreaks is important in order to understand the dynamic between the emerging strains. Hence, we followed the evolutionary dynamic of LSDV over the four years between the two outbreaks and the effect of the three-year mandatory vaccination campaign conducted post the 2019 outbreak. This study aimed to perform a whole-genome-based broad analysis of LSDV strains isolated from the 2019 and 2023 outbreaks in Israel to confirm that they have originated from the African and the Asian sources and to investigate potential recombination events between these linages.

## 2. Materials and Methods

### 2.1. Cell Line

Madin–Darby Bovine Kidney (MDBK) cells obtained from the ATCC (cell line CCL-22) were cultured in Dulbecco’s modified Eagle’s medium (DMEM, 30-2002, ATCC) supplemented with 10% Fetal bovine serum (FBS, ATCC, 30-2020), 100 units/mL penicillin, and 100 μg/mL streptomycin (Sigma-Aldrich, P4333, Saint Louis, MO, USA) and grown at 37 °C with 5% CO_2_.

### 2.2. Virus Isolation

Tissue samples from lumps extracted from sick cows that were sampled during the 2019 (Sample 354445, Kibbutz Ginosar, Israel) and 2023 (Sample 485614, Beit-Gamliel, Israel) outbreaks were homogenized in 0.5 mL PBS and filtered using a 0.45 µm filter. Next, 100 µL of the filtered fluid was added to MDBK cells in a 100 mm dish for 1 h. Then, the supernatant was aspirated and was replaced with MDBK growth medium with 5%FBS. The cells were monitored for 3 days. The cells were harvested using a cell scraper, homogenized and kept at −80 °C until use. Virus growth was confirmed using real-time PCR in comparison to the inoculated dose.

### 2.3. DNA Extraction

Viral DNA was extracted using Viral Gene-Spin Viral DNA/RNA Extraction Kit (iNtRON, JungAng Induspia V B/D, 137, Sagimakgol-ro, Jungwon-Gu, Seongnam-Si, Gyeonggi-Do, Republic of Korea) according to the manufacturer’s instructions.

### 2.4. PCR

For amplifying the 1214 bp region covering the (extracellular envelope virus) EEV glycoprotein (LSD126) for Sanger sequencing, the primers 115770Fwd (5′ACAAGGCATGTGTAGAGGCATTAG-3′) and 116984Rev (5′-GGGCATAGTAACGCTAATACATTAG-3′) were used. The reaction mixture contained 500 μg of viral DNA, 10µL of i-pfu PCR mix (iNtRON, Seongnam-Si, Republic of Korea), and 1 μL (1 pmole/µL) of each primer in a total volume of 20 μL. The reaction conditions were 95 °C for 0.5 min, followed by 40 cycles of 95 °C for 20 s, 56 °C for 10 s and 72 °C for 2 min followed by a final step of 72 °C for 5 min. The DNA products were purified and sent for Sanger sequencing with the corresponding primers [12].

### 2.5. Next-Generation Sequencing

Whole-genome sequencing (WGS) was conducted using next-generation sequencing (NGS) to determine genetic variation in DNA. After nucleic acid extraction of the isolates, LSDV_2019 and LSDV_2023 samples were sent to The Genomic Applications Laboratory, The Core Research Facility, Faculty of Medicine, The Hebrew University of Jerusalem, Israel. The DNA was quantified using the Qubit 4 system (Invitrogen, Carlsbad, CA, USA), then a DNA library was prepared using Illumina DNA Prep (Illumina DNA Prep, (M) Tagmentation (96 Samples), Illumina, Inc., San Diego, CA, USA) according to the manufacturer’s instruction. Then, the library was quantified again with the Qubit 4 system and loaded on a Nextseq500 sequencer machine (Illumina) generating 150 bp paired-end reads. Finally, the sequencing results were provided in FastQ files for the LSDV_2019 sample (LSDV_1985_19_S1_R1_001.fastq.gz) and the LSDV_2023 sample (LSDV_1364_23_S11_R1_001.fastq.gz).

### 2.6. Multiple Sequence Alignment (MSA)

All complete genome sequences of LSDV used in this project were retrieved from the NCBI public database (https://www.ncbi.nlm.nih.gov, accessed on 3 February 2025). High-quality LSDV genome sequences from the 2019 and 2023 outbreaks in Israel retrieved in this study were aligned with the LSDV genome sequences fetched from the NCBI database using the MAFFT 7.407_1 multiple alignment program (https://mafft.cbrc.jp/alignment/server/index.html (version 7.4), accessed on 3 February 2025) with the parameters of a gap extend penalty of 0.123 and a gap opening penalty of 1.53 [13].

### 2.7. Phylogenetic Analysis

IQ-TREE multicore version 1.6.12 for Windows was run locally (http://iqtree.cibiv.univie.ac.at/ (version 2016), accessed on 3 May 2025) for making the phylogenetic tree. We have applied maximum likelihood (statistical method), Bayesian information criterion (BIC), bootstrapping (UFBoot) Shimodaira–Hasegawa-like approximate likelihood ratio test RT (SH-aLRT), Bayes test and bootstrapping and nearest neighbor interchange (NNI) [14].

### 2.8. SimPlot Analysis and LAST Hit Plot

SimPlot 3.5.1 was used to measure the percent identity/similarities among the retrieved LSDV genomes and LSDV_2019 and LSDV_2023 genomes. In this study, complete genome nucleotide sequences of LSDV viruses were aligned in MAFFT 7.407_1 before they were exported to SimPlot 3.5.1 for the subsequent analysis. Then, SimPlot analysis of LSDV viruses was carried out with the Kimura (2-parameter) method by 500 base pairs of the window at a 50 base-pair step, and NCBI reference sequence NC_003027.1 (Neethling strain) was used as the query sequence. The LAST Plot hits of LSDV_ 2019, LSDV_2023, and LSDV_2012 Israel genome nucleotide sequences (GeneBank accession number KX894508.1) have been achieved using MAFFT version 7 online server with the Score = 39 (E = 8.4 × 10^11^) (https://sray.med.som.jhmi.edu/SCRoftware/SimPlot and version 3.5.1, accessed on 15 October 2025) (https://mafft.cbrc.jp/alignment/server/, accessed on 15 January 2026) [15].

### 2.9. Complete Genome Levels of Genetic Diversity

All sequences of LSDV used in this study were aligned in MAFFT 7.407_1. Then, pairwise distance analysis was performed using the MEGA X software tool (version 10, https://www.megasoftware.net/, accessed on 26 March 2026). Pairwise distance analysis was performed with the following limitations: Kimura 2-parameter model, transitions/transversions substitution, gamma distribution-shape parameter [6], and gaps/missing data were deleted pairwise. Subsequently, the exact standard errors were showed above the diagonal in the individual tables of pairwise distance analysis [16].

### 2.10. Measurement of APOBEC Motif Mutations and dN/dS Ratio

The APOBEC motif mutations in the LSDV_2019 and LSDV_2023 complete genome sequences were determined in the Hypermut 2.0 tool (https://www.hiv.lanl.gov/content/sequence/HYPERMUT/hypermutv3.html, accessed on 22 December 2025) with customized options. The LSDV_2012 genome sequence was used as the reference sequence and displayed in the respective figures. Further, the dN/dS ratio in the transcribed LSDV_2012, LSDV_2019 and LSDV_2023 genes was measured in SNAP v2.1.1 (https://www.hiv.lanl.gov/content/sequence/SNAP/SNAP.html, accessed on 22 December 2025) [17].

### 2.11. Recombination Analysis

To investigate potential recombination between the LSDV isolates from 2012, 2019, and 2023, recombination analysis was performed using the Recombination Detection Program version 4 (RDP4) (https://rdp4.software.informer.com, accessed on 11 March 2026). A statistical method, including RDP, GENECONV, BootScan, MaxChi, Chimaera, SiScan, and 3Seq, was employed to detect recombination events. The software’s internal algorithms for identifying parental lineages were cross-referenced with the chronological order of isolate collection to account for potential recombinants with high sequence similarity.

### 2.12. Measurement of Nucleotide/Amino Acid Mismatch, Transition/Transversion, and Silent/Non-Silent Mutation

The nucleotide/amino acid mismatch, transition/transversion, and silent/non-silent mutations were determined in the LSDV_2019 and LSDV_2023 genomes against the reference sequence LSDV_2012 at the complete genome levels using the Highlighter tool (https://www.hiv.lanl.gov/, accessed on 3 December 2025). This was done with or without similarity sorting of the sequences and with or without treating the gaps as a character. In addition, specific transition/transversion bias in the LSDV_2019 and LSDV_2023 genomes against the reference LSDV_2012 genome sequence was analyzed through the Highlighter tool [18]. Furthermore, non-silent mutations were analyzed through PCR to verify differences in single nucleotides of the LSDV_2019 and LSDV_2023 sequences against the reference sequence NC_003027.1 (Neethling 2490) [19]. Genes 122, 123, and 147 of LSDV_2019 and LSDV_2023 were analyzed through Chimera X software (https://www.cgl.ucsf.edu/chimerax/download.html, accessed on 4 December 2025) for structure differences, dihedral angles (φ and ψ), steric clashes, hydrogen bonding patterns and Ramachandran plot (https://www.ramplot.in (version 2025), accessed on 15 January 2026) [20].

### 2.13. Promoter Motif and RNA Secondary Structure Prediction

Two DNA sequences corresponding to the upstream region of LSDV gene 23 were analyzed: LSDV_2019 and LSDV_2023. These sequences were obtained from previously annotated LSDV genomic data and aligned to the NC_003027 (NI_2490) reference genome using NCBI BLAST to confirm their genomic context and orientation relative to gene 23.

To identify motif positions, consensus sequences and binding scores in gene 23 of both LSDV_2019 and LSDV_2023, the sequences were analyzed using the MEME suite and STREME tool (https://meme-suite.org/meme/tools/meme (version 5.5.9), accessed on 22 December 2025). DNA sequences were converted to RNA by replacing thymine (T) with uracil (U) prior to secondary structure prediction. RNA secondary structures were predicted using the RNAfold Web Server (http://rna.tbi.univie.ac.at/cgi-bin/RNAWebSuite/RNAfold.cgi, accessed on 22 December 2025), part of the ViennaRNA Package 2.0. This tool calculates the minimum free energy (MFE) structure and base-pairing probabilities using dynamic programming algorithms.

### 2.14. Relative Synonymous Codon Usage Analysis

Relative synonymous codon usage (RSCU) analysis provides a ratio between the observed and expected value of synonymous codons for particular amino acids in the gene. There are some RSCU values which indicates codon biases: 1 shows no codon bias for that specific codon, more than 1 denotes positive codon usage bias (defined abundant codons), and less than 1 suggests negative codon usage bias (defined less-abundant codons) [21]. In this study, the RSCU values for the LSDV_2012, LSDV_2019 and LSDV_2023 genome sequences were determined using CodonW 1.4 software (https://codonw.sourceforge.net, accessed on 28 December 2025).

### 2.15. Effective Number of Codons

The effective number of codons (ENc) estimates how many of the 61 codons are actually used to encode the 20 amino acids with values ranging from 20 to 61, where an ENc value higher than 50 shows weak bias in codon usage whereas lower than 35 shows strong bias in codon usage [22]. In the present study, the ENc values of genes from the LSDV_2012, LSDV_2019 and LSDV_2023 genome sequences were evaluated using CodonW 1.4 software (https://codonw.sourceforge.net, accessed on 28 December 2025).

### 2.16. Codon Adaptation Index

The codon adaptation index (CAI) determines the similarity of synonymous codon usage bias between a query gene and the reference gene. Generally, CAI values range from 0 to 1, in which a value close to 1 shows strong or high bias of codon usage or protein expression and vice versa [23]. The coding region nucleotide sequences of LSDV_2012 genes were retrieved from the NCBI nucleotide public database. Then, the CAI values of LSDV_2019 and LSDV_2023 genes were evaluated using Galaxy bioinformatics tools (https://usegalaxy.eu, accessed on 28 December 2025).

### 2.17. ENc-GC3s Plot

The ENc-GC3s plot determines whether the codon usage bias of specific genes is affected by selection pressure or mutation pressure. These analyses are visualized using a plot between the ENc values against the GC content at the third codon positions (GC3s). When the codon usage bias of a specific gene is affected by selection pressure, then the ENc-GC3 plot value falls below the expected curve, whereas when the codon usage bias of a specific gene is affected by mutation pressure, the ENc-GC3 plot value lies on or around the expected curve [24]. The ENc value and GC3 value for LSDV_2019 and LSDV_2023 against LSDV_2012 were evaluated through CodonW 1.4 software, and graphs were created through Sigmaplot software (https://codonw.sourceforge.net, accessed on 28 December 2025).

### 2.18. Neutrality Plot Analysis

The intensity of mutation pressure and natural selection on codon usage patterns are determined by GC12 values plotted against GC3 values of the codon in the neutrality plot. A scatter plot was created and linear regression was executed using GC3 as the x-axis and GC12 as the y-axis for each genome. If a scatter point falls close to the diagonal line, and the values of GC12 and GC3 are similar, there are small differences in the base composition of the codons, suggesting that the codon bias is induced by mutation, and vice versa for selection pressure [25]. The GC12 and GC3 values for the nucleotide sequences of LSDV_2019 and LSDV_2023 against LSDV_2012 were estimated using CodonW 1.4 software (https://codonw.sourceforge.net, accessed on 28 December 2025).

### 2.19. PR2 Plot Analysis

A PR2 plot was used to evaluate the relative influences of natural selection and mutation pressure to the codon usage bias in the LSDV_2019 and LSDV_2023 genome sequences. The PR2 plot imitates the base composition of the third nucleotide in the codon. The x-axis shows the G3/(G3 + C3) ratio and the y-axis shows the A3/(A3 + T3) ratio for each gene. Thus, the scatter plot that was created and the unbiased condition of a codon are represented at the center, where the direction and the distance of the scatter point relative to the center point correlates to the bias of the gene (https://codonw.sourceforge.net, accessed on 28 December 2025) [26].

## 3. Results

### 3.1. Phylogenetic Analysis of EEV Gene of LSDV Isolates from 2019 and 2023 Outbreaks

**Figure 1:**
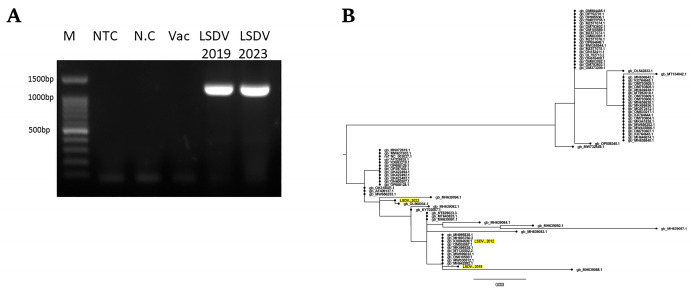
Amplification of LSDV EEV gene and phylogenetic analysis. (**A**) PCR results of 1214 bp fragment amplification by PCR on the LSDV DNA. M: 100 bp ladder; NTC: non-template control; N.C: negative control—skin biopsy from non-infected cow; Vac: vaccine strain control, LSDV_2019 and LSDV_2023. (**B**) Phylogenetic analysis of the 1214 bp fragments of LSDV_2019 and LSDV_2023 in relation to selected LSDV corresponding fragments taken from NCBI. The genome labels represent the gene bank accession number of each sequence. Israeli isolates from outbreaks in 2012, 2019 and 2023 are highlighted in yellow (the original PCR picture can be found in Figure S3).

LSDVs from the 2019 and 2023 outbreaks in israel were isolated on MDBK cells as previously described [27]. The TCID_50_ of each strain was estimated, followed by DNA extraction and amplification of the EEV gene as described in Materials and Methods (Figure 1A). Following Sanger sequencing, the sequences were aligned to the corresponding fragment of LSDV sequences published in the NCBI database (Table S1). We estimated the origin of each sample by building a phylogenetic tree of the samples with published LSDV sequences of the same fragment (Figure 1B). The results show that the LSDV_2019 sample which originated from the north-east border was closely related to an LSDV sample from 2015 (RNOA-15) from North Ossetia-Alania, Russia (GB accession number: MH077562), suggesting a North Asian origin. However, the LSDV_2023 sample which originated from the south-west border was closely related to an LSDV sample from the 2021 outbreak in El-Sharkia, Egypt (GB accession number: OL960034). Based on these results, we progressed to whole-genome sequencing in order to trace any genomic changes in the LSDV_2023 genome in comparison to the LSDV_2019 genome and see whether recombination events had occurred during the four years in between the outbreaks.

### 3.2. Whole-Genome Phylogenetic Analysis

**Figure 2:**
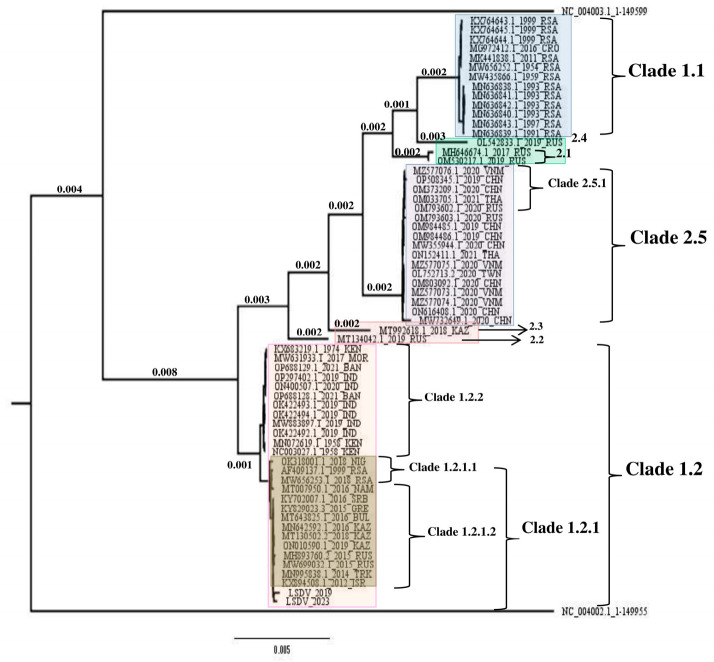
Phylogenetic analysis of selected whole LSDV genomes. Selected whole LSDV genomes (Supplementary Table S2) and assembled genomes of isolates from the outbreaks in Israel in 2019 and 2023. All LSDV genomes were aligned in MAFFT 7.407_1 software and the phylogenetic tree was made using IQ-TREE software.

To determine the phylogenetic relationship between the complete LSDV genomes from the 2019 and 2023 outbreaks in Israel, we performed whole-genome next-generation sequencing (WGS) on the isolates from both outbreaks as described in Material and Methods. Next, multi-sequence alignment (MSA) was done on selected LSDV full genome sequences from the NCBI public database (Supplementary Table S2) and the LSDV_2019 and the LSDV_2023 genomes followed by phylogenetic analysis. The analysis showed that both the LSDV_2019 and the LSDV_2023 sequences clustered to a common sub-clade, 1.2.1, with proximity to the 1.2.1.2 sub clade. This sub-clade, which includes isolates from South Africa and from Europe/Kazakhstan, also includes the LSDV isolate from the 2012 outbreak in Israel, which represents the Pan-African/Eurasian isolates as described previously [28]. Hence, one can estimate that both the LSDV_2019 and the LSDV_2023 isolates relate to the LSDV sequences isolated from the South African strains or from the European/Kazakh strains and might be related to the LSDV_2012 isolate (Figure 2). This analysis supports the assessment that the LSDV strains from 2012, 2019 and 2023 outbreaks in Israel are phylogenetically related and might have originated from a South African or from a North Asian sub-clade.

### 3.3. SimPlot Analysis and LAST Hit Plot

**Figure 3:**
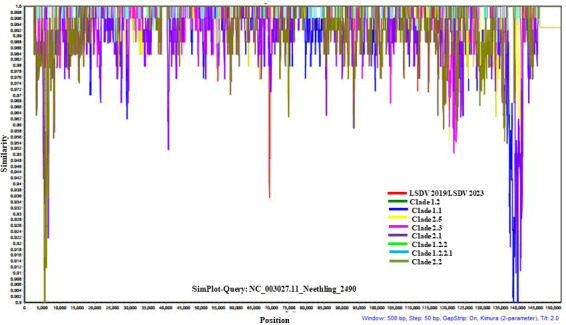
SimPlot analysis of gnomic gaps between LSDV isolates. The SimPlot analysis of the genomic gaps among all LSDVs at the whole-genome nucleotide sequence levels. Similarity values are detailed in the y-axis. The Neethling strain NC_003027.1 genome was used as a reference in this analysis.

Previous studies using SimPlot analysis have shown that LSDV isolates exhibit low genetic diversity [29]. Similarly, the analysis that was done in the present study had shown that the genetic diversity between LSDV_2019 and LSDV_2023 genomes is low, considering the Neethling_2490 genome as the reference genome (Figure 3 and Supplementary Table S2). This analysis showed that the LSDV_2019 and the LSDV_2023 sequences contain single nucleotide variations and some transition/transversion mutations. Moreover, there are inter-genomic deletions of 17 nucleotides found between genes 22 and 23 in LSDV_2023 in comparison to the corresponding region in LSDV_2012 and in LSDV_2019. In addition, LAST hit plot results confirmed that LSDV_2019 and LSDV_2023 sequences have 38 single nucleotide deletions, while there are 49 single nucleotide deletions between LSDV_2012 and LSDV_2023 (Supplementary Figure S1).

### 3.4. Complete Genome Levels of Genetic Diversity and Measurement of APOBEC and dN/dS Ratio

Complete genome levels of genetic diversity analysis was used to identify the genetic diversity at the genome level over the years between the outbreaks in Israel using the LSDV_2012, LSDV_2019 and LSDV_2023 isolates. This analysis revealed that LSDV_2019 has 0.05% and 0.34% genetic diversity at the genome level with LSDV_2023 and LSDV_2012, respectively. Similarly, LSDV_2023 has 0.06% diversity with LSDV_2019 and 0.25% diversity with LSDV_2012 (Table 1). These results indicate that while both LSDV_2019 and LSDV_2023 are closely phylogenetically related to LSDV_2012, the genomic similarity between LSDV_2019 and LSDV_2023 is higher, suggesting an evolutionary relationship between all three isolates but an even closer one between the two isolates described in this study.

**Figure 4:**
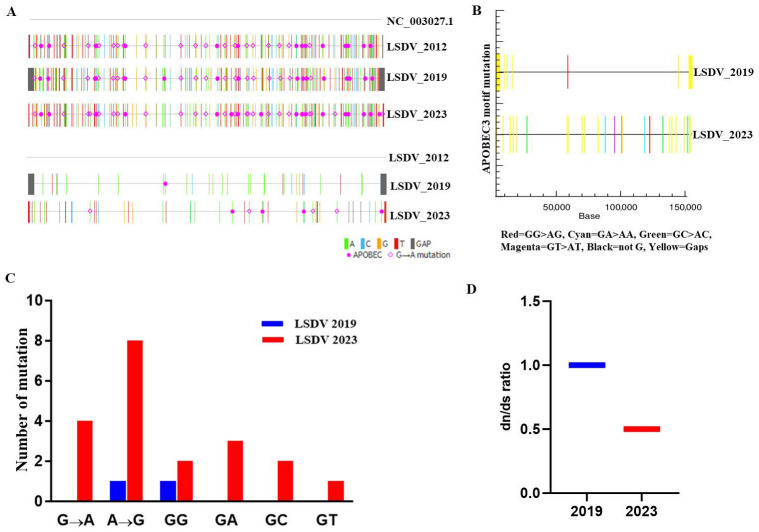
APOBEC Mutations and dN/dS ratios of LSDV isolates. (**A**) The APOBEC3 mutations analysis exposed the enrichment of APOBEC3 mutations in LSDV_2019 and in LSDV_2023 genomes using LSDV_2012 as a reference genome. (**B**) The APOBEC3 motif mutations represented in LSDV_2019 and LSDV_2023 genomes using LSDV_2012 genome as a reference. (**C**) Graphical representation showing different types of APOBEC3 and non-APOBEC3 motif mutations in LSDV_2019 and LSDV_2023 genomes using LSDV_2012 genome as a reference. (**D**) Graphical representation showing dN/dS ratios of LSDV_2019 and LSDV_2023 genomes using LSDV_2012 genome as a reference.

Differences observed in the APOBEC genes among LSDV_2012, LSDV_2019, and LSDV_2023 are attributable to nucleotide transitions and transversions. In this study, it was found that APOBAC3 activity mutations are more frequent in LSDV_2023 where guanine → adenine transition is more than in LSDV_2019 at the genome level (Figure 4A,B). Moreover, LSDV_2023 showed more G → A, GA > AA and GG > AG motif mutations than LSDV_2019, as compared to LSDV_2012, which suggests that the 2023 strain of the virus has been edited by the host’s immune system much more heavily than the 2019 strains (Figure 4C and Supplementary Data S1). In addition, these results suggest that LSDV 2019 has very few changes in G → A (relatively similar to the older version), whereas LSDV 2023 shows more G → A, GA > AA and GG > AG mutation over a short period of time; thus, the hosts’ immune systems are reacting more aggressively toward it. In addition, when we compare the dN/dS ratio of LSDV_2019 and LSDV_2023 with LSDV_2012, the ratios were found to be 1.0 and 0.50, respectively. Consequently, these ratios indicate that LSDV_2019 showed neutral evolution, whereas LSDV_2023 has negative/purifying selection (Figure 4D and Data S1). All these results indicate that LSDV_2023 exhibits a higher number of APOBEC-associated mutations, primary driven by transition events which helped cause G → A and C → T errors in DNA replication or altered RNA function in comparison to LSDV_2019.

### 3.5. Recombination Analysis

Recombination analysis of the LSDV genomes using RDP4 software discovered a single recombination signal consistently detected by four independent RDP4 methods—BootScan, MaxChi, Chimaera, and 3Seq—each producing highly significant *p*-values (ranging from 6.38 × 10^−5^ to 2.13 × 10^−3^). Thus, the results strongly support the presence of a genuine recombination event in the dataset. On the contrary, other algorithms such as RDP, GENECONV, SiScan, PhylPro and LARD, did not detect any recombination events (Table 2). Yet, this pattern is common due to differences in algorithm sensitivity, and the agreement among multiple methods is generally considered robust evidence.

**Table 2:** RDP4 recombination analysis of LSDV genome.

S. No	Method	Sequence Detected in	Average *p*-Value
1	RDP	0	0
2	GENECONV	0	0
3	BootScan	1	1.498 × 10^−3^
4	MaxChi	1	6.381 × 10^−5^
5	Chimera	1	1.829 × 10^−4^
6	SiScan	0	0
7	PhylPro	0	0
8	LARD	0	0
9	3Seq	1	2.130 × 10^−3^

### 3.6. Measurement of Nucleotide/Amino Acid Mismatch, Transition/Transversion, and Silent/Non-Silent Mutation

**Figure 5:**
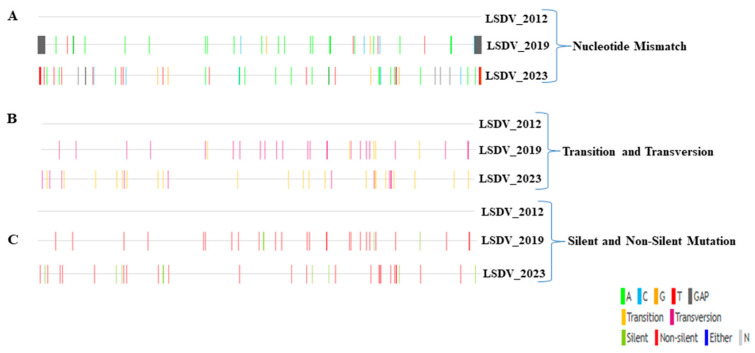
Genomic comparison of nucleotide mismatch, transitions/transversions and mutations between Israeli LSDV isolates. (**A**) The nucleotide mismatch analysis displays the presence of mismatches in the LSDV_2019 and LSDV_2023 genomes in reference to the LSDV_2012 genome. (**B**) The nucleotide transition and transversion analysis exhibited in the 2019 and 2023 genomes and the NC_003027.1 sequence was used as a reference in this analysis. (**C**) The silent and non-silent mutation analysis showed the enhancement of non-silent mutations in the 2019 and 2023 genomes and the NC_003027.1 sequence was used as a reference in this analysis.

Other notable differences between the LSDV_2019 and LSDV_2023 genomes may also provide valuable insights into the evolutionary dynamics of this variant. Both LSDV_2019 and LSDV_2023 exhibited several nucleotide/amino acid mismatches, as shown in Figure 5. When compared to LSDV_2012, LSDV_2019 has more transversions (43%) than transitions (8%), which indicates that the mutation profile is dominated by transversions. On the contrary, LSDV_2023 presented more transitions (25%) than transversions (14%), when compared to LSDV_2012 (Figure 5 and Supplementary Data S2).

The dominance of transversions in LSDV_2019 compared to LSDV_2012 suggests that the virus was under strong mutagenic or immune-driven pressure during its rapid geographic expansion, leading to more disruptive nucleotide changes. In contrast, the higher proportion of transitions in LSDV_2023 indicates a shift toward more conservative, polymerase-driven mutations that typically arise during stable viral circulation. This pattern implies that LSDV_2023 experienced reduced selective pressure and may be better adapted to its host environment. Overall, the shift from transversion- to transition-dominated mutations reflects a transition from active adaptation to evolutionary stabilization in the virus. Overall, both the LSDV_2019 and the LSDV_2023 genomes exhibit various genetic differences relative to LSDV_2012. These differences include nucleotide mismatches, point mutations (transitions and transversions), and non-silent mutations that may influence viral evolution and protein function (Supplementary Data S2 and Figure S2).

**Figure 6:**
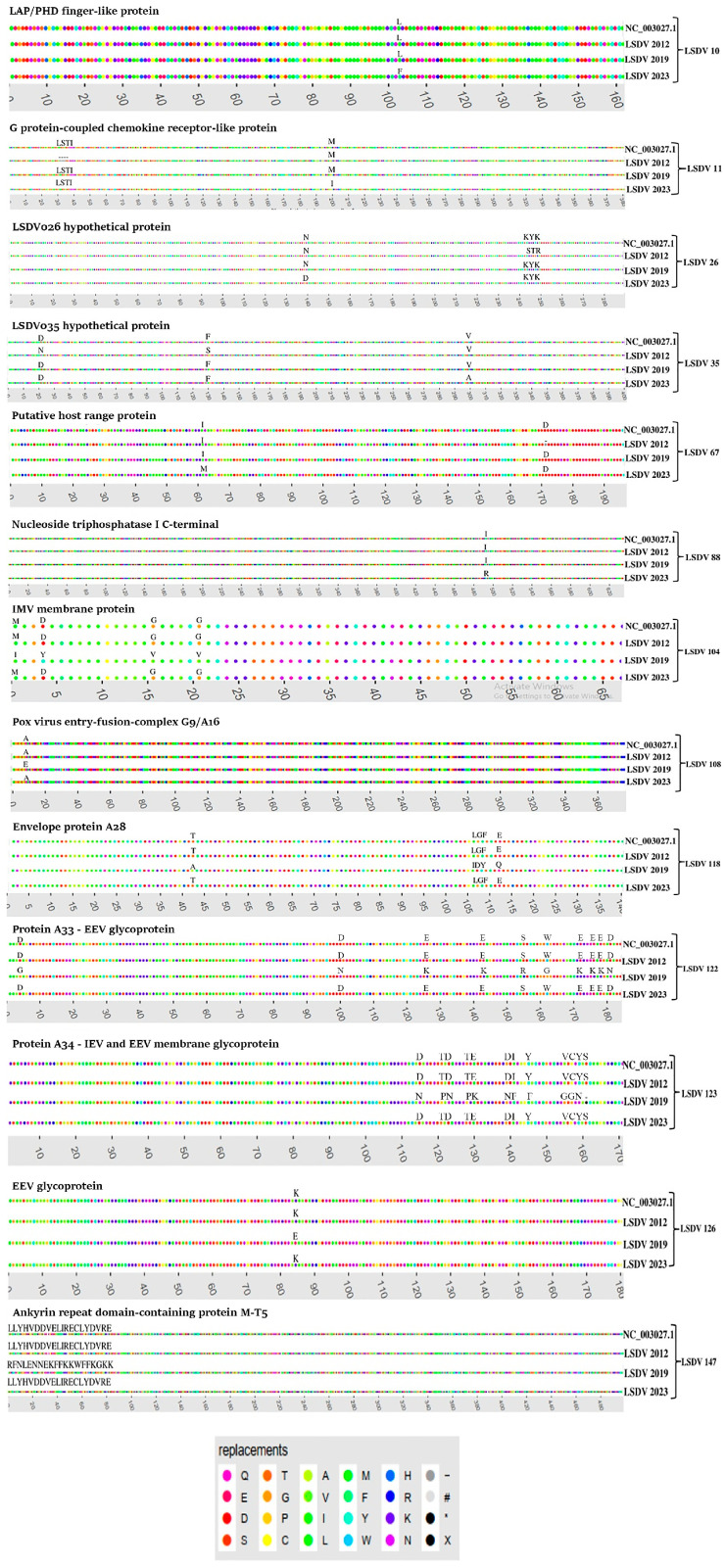
Non-silent mutations in LSDV_2012, LSDV_2019 and LSDV_2023 proteins. The non-silent mutations in LSDV_2012, LSDV_2019 and LSDV_2023 were explored via the Highlighter bioinformatics tool in reference to the Neethling strain (NC_003027.1) and validated by PCR and Sanger sequencing. Sequence alignments are presented for protein products of LSDV genes 10, 11, 26, 35, 67, 88, 104, 108, 118, 122, 123, 126, 127, and 147. The changes between specific AAs are presented via the Variant visualizer tool.

Subsequently, we further characterized the non-synonymous mutations that were found in LSDV genes 10, 11, 26, 35, 67, 88, 104, 118, 121, 122, 123, 126, 127 and 147, when comparing the LSDV_2019 and LSDV_2023 isolates. These mutations were confirmed by PCR and Sanger sequencing of the relevant regions (Table S3), followed by using the Variant visualizer and the Expasy Translation tool to identify the resulting single amino acid substitutions (Figure 6 and Supplementary Table S4).

Interestingly, genes 10, 11, 26, 35, 67, 88, 108 and 118 in LSDV_2019 are similar to the corresponding genes of LSDV_2012, with the exception of three amino acids (serine–threonine–arginine) that are located in proximity to the N-terminus of gene 26, which were replaced with lysine–tyrosine–lysine in LSDV_2023 and in Neethling (NC_003027.1). However, for genes 104, 122, 123, 126 and 147, there is higher similarity in LSDV_2023 to LSDV_2012. These changes between LSDV_2019 and LSDV_2023 in comparison to LSDV_2012 and to the vaccine strain Neethling suggest possible recombination events that occurred between LSDV_2012 or Neethling with LSDV_2019 during the years between the outbreaks that have produced the LSDV_2023 variant.

### 3.7. Structural Analysis of Protein Products of LSDV Genes 122, 123 and 147

**Figure 7:**
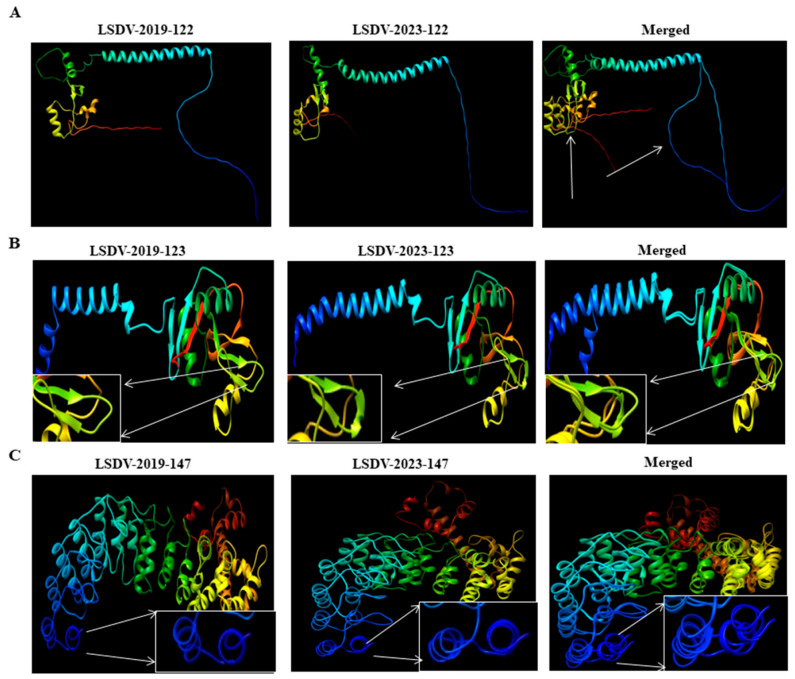
Comparison of predicted LSDV protein structures. Predictions of protein structures of LSDV_2019 variants (left panel), LSDV_2023 variants (middle panel) and merged structures (right panel) are presented for protein 122 (**A**), protein 123 (**B**) and protein 147 (**C**). Alterations in the structures between variants are presented through the Chimera X (Version 1.10.1) protein structure tool and marked with arrows.

In particular, we have focused on genes 122, 123 and 147 for the structural analyses. These genes were selected since they are known to be involved in various crucial functions in LSDV, and a higher number of non-silent mutations was identified than in the other listed genes. Specifically, we focused on three proteins: (1) protein A33, the product of gene 122, that was suggested to be involved in formation of the intermediate virions; (2) protein A34, the product of gene 123, that is involved in intercellular and extracellular enveloped virus (EEV) production, both playing an essential role in efficient cell-to-cell spread of viral particles; and (3) the protein product of gene 147 that encodes a B4R-like ankyrin repeat protein (ANK repeat protein), a paralog to a vaccinia virus protein (C9L) that mediates diverse protein–protein interactions. For structural characterization, we generated models of the protein products of genes 122, 123 and 147 of LSDV_2019 and LSDV_2023 using Chimera X. Sequence-driven alterations were detected in the predicted structure of the LSDV_2019 strain in comparison to the corresponding protein in the LSDV_2023 isolate (Figure 7).

Subsequently, we used these models for comparative analyses of the backbone dihedral angles (φ and ψ), steric clashes and hydrogen bonding patterns between each of the protein variants identified in LSDV_2019 and in LSDV_2023. Distinct conformational differences may underlie structural divergence. In the protein A33, the structure of the variant from LSDV_2019 displayed several residues with φ/ψ values characteristic of a stable α-helical or extended conformation, accompanied by moderate to high hydrogen-bond counts, such as Asn100 (13 H-bonds) and Lys143 (26 H-bonds). In contrast, the LSDV_2023 variant showed altered φ/ψ distributions, including residues such as Glu126 and Glu176 adopting more extended or strained conformations, often accompanied by reduced hydrogen bonding. Notably, Arg155 in the LSDV-2019 variant exhibited an exceptionally high clash score (1324), whereas the corresponding Ser155 in the LSDV-2023 variant showed few clashes, suggesting a major shift in local packing and steric environment. In protein A34, both variants exhibited residues occupying extended β-strand regions; however, the magnitude of steric strain and hydrogen-bonding differed markedly. While the LSDV_2019 variant showed strong stabilization at Asn115 (44 H-bonds) and moderate clashes at Lys129 (291 clashes), the LSDV_2023 variant demonstrated altered φ/ψ values and a redistribution of stabilizing interactions. These differences suggest that protein A34 in LSDV_2023 adopts a more strained and less uniformly stabilized conformation compared to its LSDV_2019 counterpart.

The most pronounced structural divergence was observed in the ANK repeat protein, the protein product of gene 147. The LSDV_2019 variant displayed several residues with stabilizing hydrogen-bond networks, such as Glu14 (42 H-bonds), Asn32 (23 H-bonds), and Lys20 (16 H-bonds), consistent with well-packed and energetically favorable conformations. In contrast, the LSDV_2023 variant exhibited widespread increases in steric clashes, including extreme values at Arg24 (379 clashes) and Arg55 (785 clashes). Several residues also showed reduced or no hydrogen bonding, such as Val14 and Cys34. Shifts in φ/ψ angles were also evident, with several residues transitioning from α-helical regions in the LSDV_2019 variant to extend or less favorable regions in the LSDV_2023 variant.

**Figure 8:**
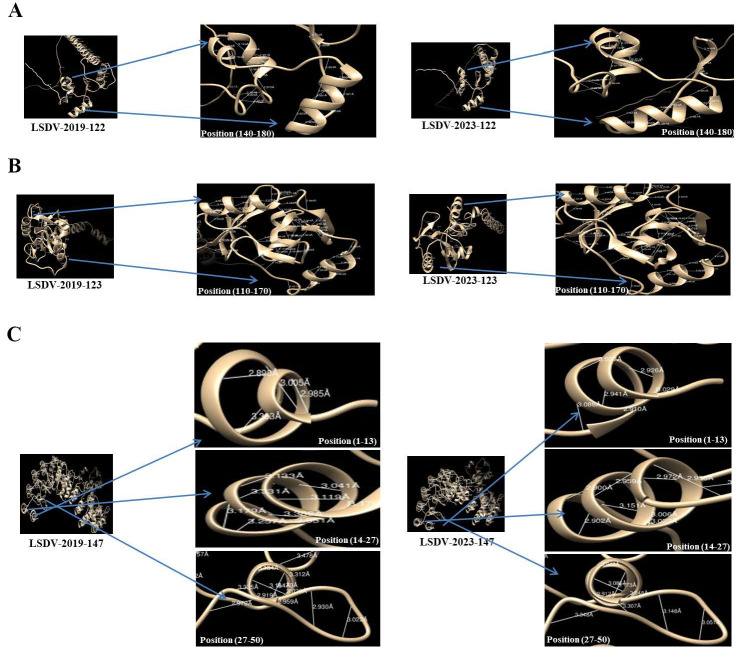
Measurements of protein structure and hydrogen-bonds of protein products from LSDV_2019 and LSDV_2023 isolates. Measurement of protein structure variants LSDV_2019 (**left panel**) and LSDV_2023 (**right panel**) of gene 122 (**A**), gene 123 (**B**) and gene 147 (**C**). Alteration in phi/psi values and distinct hydrogen-bond distances are presented through the Chimera X protein structure tool.

The hydrogen bond distances across the structures of the protein products of genes 122, 123 and 147 from LSDV_2019 and LSDV_2023 were compared. These analyses revealed notable differences in bonding geometry and stabilization. In comparison to the LSDV_2023 protein variants, in the LSDV_2019 protein variants, the hydrogen bonds were consistently shorter and more uniformly distributed, particularly in regions spanning residues 140–180 in protein A33 and residues 110–170 in protein A34. In contrast, the LSDV_2023 variants exhibited longer and more variable hydrogen bond distances, with several interactions falling near or beyond the upper threshold for stabilizing contacts. Furthermore, it was especially evident in the LSDV_2023 ANK repeat protein variant, where the regions spanning residues 1–50 showed reduced hydrogen bond density and increased spatial separation between donor and acceptor atoms (Figure 8 and Table S5).

**Figure 9:**
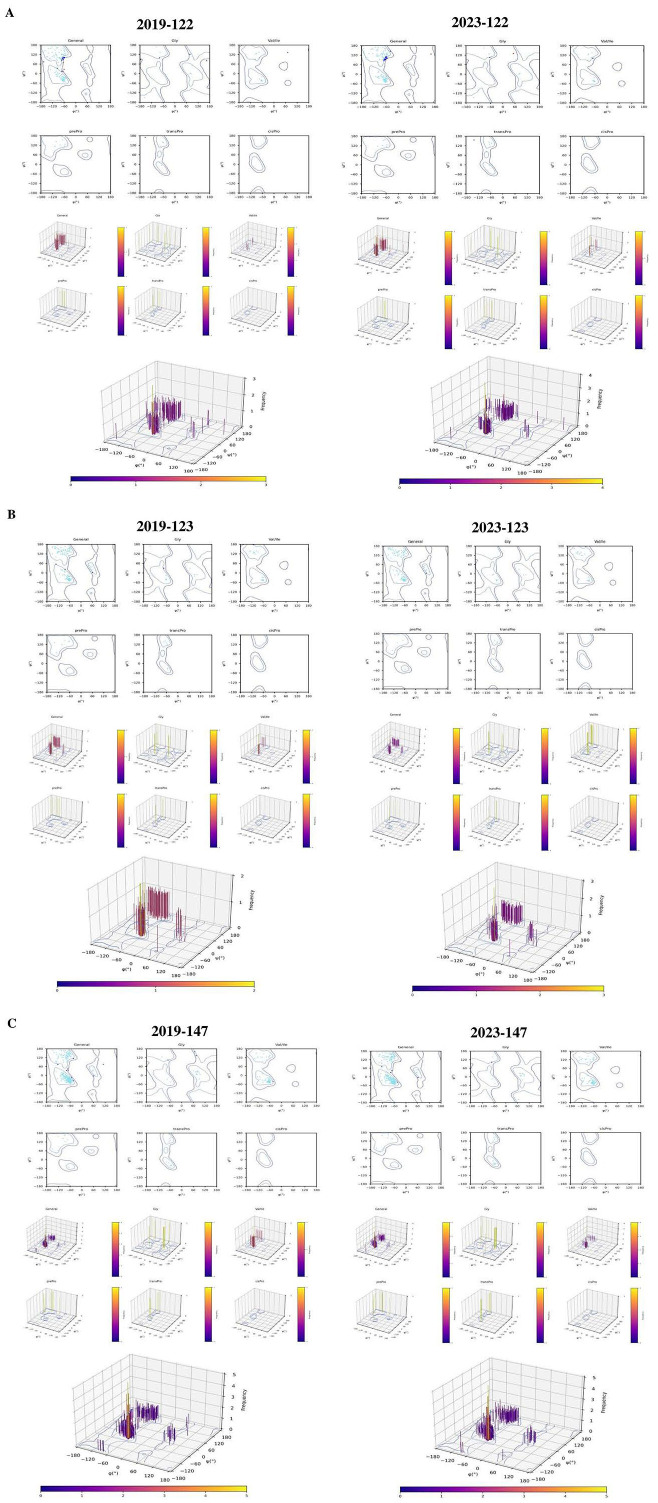
Ramachandran plot analysis of protein products of LSDV variants. (**A**) Amino acid differences were analyzed through Ramachandran plot and representation of glycine, valine, pre-proline, trans-proline, and cis-proline amino acids in 2D plot. (**B**) Representation of glycine, valine, pre-proline, trans-proline, and cis-proline amino acids in 3D plot. (**C**) Representation of all amino acids in 3D plot.

Finally, Ramachandran plot analysis of the protein products of genes 122, 123 and 147 from the LSDV_2019 and LSDV_2023 isolates revealed clear differences in the backbone conformation between the two variants. In all three proteins, the LSDV_2019 variant structures showed that the majority of the residues clustered within the favored α-helix and β-strand regions, indicating stable and energetically favorable backbone geometry. In contrast, the LSDV_2023 protein variants displayed a broader and more scattered distribution of φ/ψ angles, with several residues occupying less-favored or outlier regions of the Ramachandran map. Overall, the Ramachandran plots indicate the conclusion that the LSDV_2023 protein variants adopt more strained and less stable backbone conformations compared with the structurally more ordered LSDV_2019 protein variants (Figure 9).

### 3.8. Motif Analysis and RNA Secondary Structures of Inter-Genomic Region of LSDV Gene 22–23

Another intriguing genomic difference between the LSDV_2019 and the LSDV_2023 isolates was a 17 nucleotides deletion (AGTTATAGTAGTATTAT) in the inter-genomic region between gene 22 and gene 23 found in LSDV_2023. Interestingly, we identified that the inter-genic deletion in LSDV_2023 was unique and did not match with other LSDV strains circulating in the region. The MEME suite, including STREME tools, was used to perform motif analysis of the inter-genomic region between gene 22 and gene 23 of LSDV_2019 and LSDV_2023 to identify conserved regulatory elements.

**Figure 10:**
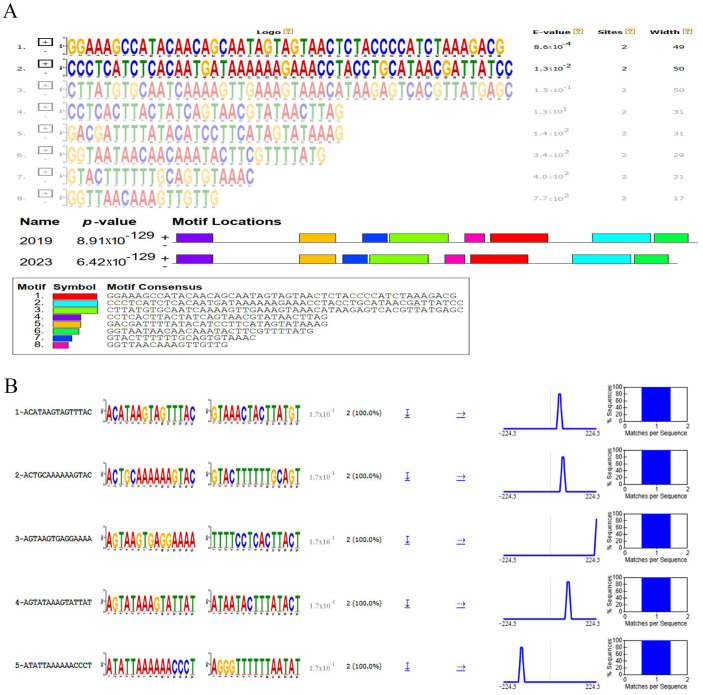
Sequence motif analysis of inter-genomic region of gene 23. (**A**) Motif discovery in LSDV of gene 23 with inter-genomic region via MEME suite presents similar motifs in both LSDV_2019 and LSDV_2023 sequences. Motif locations are represented in the lower panel. (**B**) STREME analysis of five significantly enriched motifs that were identified in the LSDV_2019 and LSDV_2023 sequences. Motif names are approximations based on IUPAC consensus sequences and ranked by statistical significance. The “Logo” and “RC Logo” columns display the sequence logos for the forward and reverse-complement strands. The score indicates the statistical significance (*p*-value or E-value) of motif enrichment. Positional distribution plots show the frequency of motif occurrences relative to the sequence centers. Matches-per-sequence histograms illustrate the distribution of the number of motif matches found across the analyzed sequence.

MEME suite analysis revealed that the inter-genomic region upstream to gene 23 of both LSDV_2019 and LSDV_2023 has the same motif sequences, but with a shift in their location corresponding to the 10 bp deletion, as shown in Figure 10A. Furthermore, STREME analysis has shown a conserved motif present in both sequences with identical positional distribution and frequency. Each motif exhibited a highly significant E-value (1.7 × 10^−1^), indicating a strong enrichment and suggesting potential binding of transcription factors (Figure 10B). However, besides the 10 bp deletion, no variations in the motif sequences, relative locations or abundances were observed when comparing the inter-genomic region sequences of LSDV_2019 and LSDV_2023, implying that these transcriptional regulatory elements are conserved.

**Figure 11:**
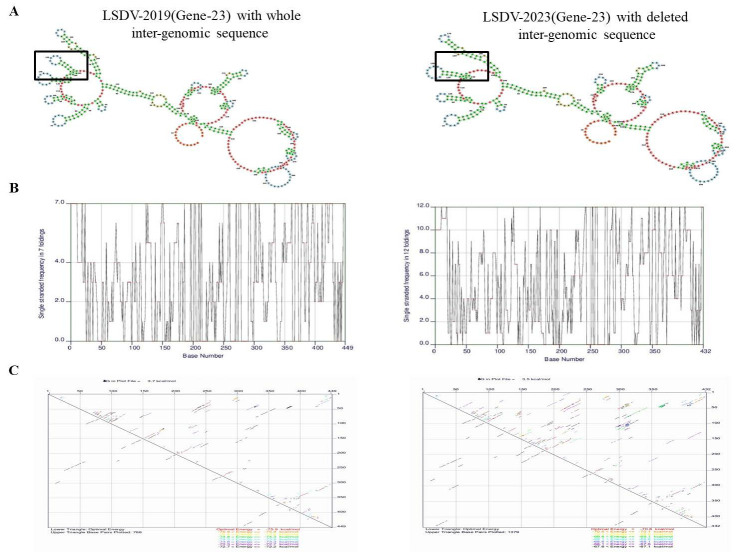
(**A**) RNA secondary structure of LSDV of gene 23 with inter-genomic region showed structure difference in LSDV_2019 and LSDV_2023 sequences via RNAFold software. (**B**) Single-stranded frequency of LSDV_2019 and LSDV_2023 was analyzed through RNAFold. (**C**) Minimum free energy (MFE) of LSDV_2019 and LSDV_2023 was again analyzed through RNA Fold.

To further assess the functional impact of this deletion, we predicted the RNA secondary structures of the inter-genomic region between genes 22 and 23, using the RNAfold web server. This sequence from LSDV_2019 produced a stable RNA structure with thermodynamics or minimum free energy (MFE) of −75.9 kcal/mol, forming multiple stem-loops and hairpins that may regulate translation and RNA stability. On the contrary, the sequence from LSDV_2023 produced a less stable structure with an MFE of −70.6 kcal/mol, lacking key folding domains and showing reduced structural complexity (Figure 11). These differences indicate that the LSDV_2019 transcript in this genomic region is thermodynamically more stable, potentially enabling efficient translation of gene 23. In contrast, the LSDV_2023 variant appears structurally compromised. These findings suggest that post-transcriptional regulations may play a role in transcriptional control, driving the observed biological differences.

### 3.9. Relative Synonymous Codon Usage and Codon Adaptation

**Figure 12:**
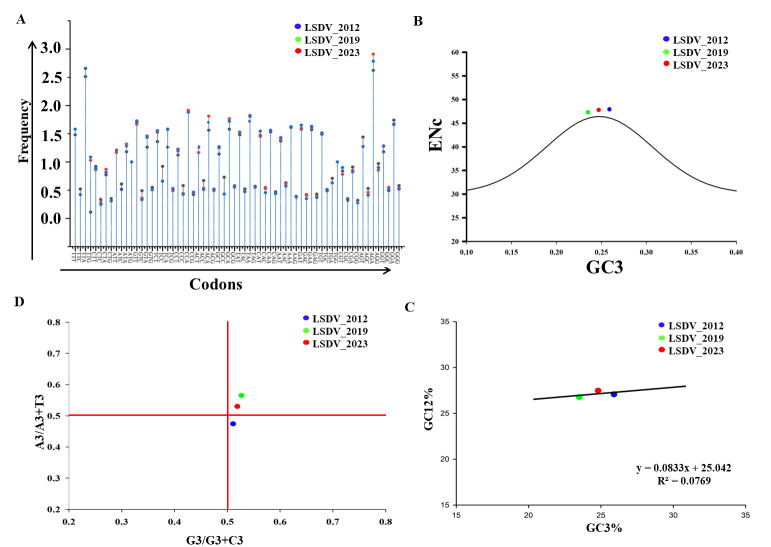
Codon usage analysis of whole genome of Israeli isolates. (**A**) Comparative analysis of relative synonymous codon usage (RSCU) patterns between LSDV_2012, LSDV_2019 and LSDV_2023. The x-axis represents the codons, while the y-axis represents the frequency. (**B**) ENc-GC3s plot values for genes that displayed mutations pressure in LSDV_2012, LSDV_2019 and LSDV_2023 genomes. (**C**) Neutrality plot between (GC3 vs. GC12) for the entire coding sequence of LSDV_2012, LSDV_2019 and LSDV_2023 genomes. GC12 represents GC at the 1st and 2nd codon positions, while GC3 represent GC at the 3rd codon position, and the black solid line represents the regression analysis of GC12 against GC3. (**D**) Parity rule (PR2) plot analysis of all three LSDV genome. The horizontal and vertical axes represent GC3/(G3 + C3) and A3/(A3 + T3), respectively. In all panels, LSDV_2012, LSDV_2019 and LSDV_2023 are shown in blue, green and red colors, respectively.

Relative synonymous codon usage (RSCU) analysis can be used as a powerful tool to track evolutionary changes over time between outbreaks. Therefore, we performed RSCU analysis to follow the nucleotide variation between the LSDV isolates. Initially, RSCU analysis revealed that all 18 possible codons were used abundantly in both the LSDV_2019 and in LSDV_2023 genomes, similarly to the usage in LSDV_2012 [TTT (Phe), TTA (Leu), ATT (Ile), GTT (Val), AGT (Ser),CCT (Pro), ACT (Thr), GCT (Ala), CAT (His), TAT (Tyr), CAA (Gln), AAT (Asn), AAA (Lys), GAT (Asp), TGT (Cys), CGT (Arg), and GGT (Gly)]. The distribution of A or T at the end codon bases was also higher than that of G or C codon bases in the genome of both LSDV_2019 and LSDV_2023. Moreover, most codons ending in A or T were overrepresented (>1.5), while those ending in G or C were underrepresented (RSCU values higher than 0.5 and less than 1). This indicates that mutational pressure was the main driver of evolution in both the LSDV_2019 and LSDV_2023 variants (Figure 12A and Supplementary Table S6).

Further, the codon adaptation index (CAI) was used to analyze codon usage bias of the viral genomes of LSDV_2012, LSDV_2019 and LSDV_2023, as well as their adaptation to the host. When comparing the LSDV isolates from the 2012, 2019 and 2023 outbreaks in Israel, all three LSDV genomes have CAI values that range between 0.2 and 0.3, which is lower than 0.5. Hence, all three LSDV genomes presented lower codon usage value than the bovine host codon usage value [30]. Next, the values of the effective number of codon (ENc) usage bias within LSDV coding sequences was calculated (Figure 12B and Supplementary Data S3). This calculation showed that the ENC values of LSDV_2019, LSDV_2023 and LSDV_2012 were 47.31, 47.80 and 47.92, respectively, indicating that the usage differences of all strains are moderate. In addition, all the strains’ values lie on the expected curve, indicating a mutational pressure on the codon usage pattern. Hence, this data suggests that ENc values and codon usage of the LSDV strains from the 2012, 2019 and 2023 outbreaks have low to moderate codon bias, as also observed in other LSDV isolates [31].

Neutrality plot evaluation was performed to show the connection between guanine/cytosine at positions 1 and 2 (GC12) and GC3 and to provide information on mutational pressure and selection pressure on the genes. The plot analysis showed that the slope of the regression line was 0.0833 (Y = 0.0833X + 25.042, R2 = 0.0769, *p* value < 0.0001). According to relative neutrality, this equation suggest that mutation pressure causes only 8.3% (0.083 × 100) of the codon usage patterns, in comparison to selection pressure, which has 91.7% in both the LSDV 2019 and LSDV_2023 genomes, considering LSDV_2012 as reference genome (Figure 12C and Supplementary Data S3). This result indicates that the LSDV outbreaks in 2019 and in 2023 occurred due to both mutational and selection pressure.

In addition, we used PR2 plots as complementary approach to identify whether codon usage of each strain is influenced by mutation pressure or natural selection pressure. The observed PR2 values of the LSDV_2019 and LSDV_2023 genomes fall within the upper right quadrant, while the PR2 value of LSDV_2012 falls within lower right quadrant. These differences indicate that in LSDV_2019 and in LSDV_2023, biological mutational pressure is more influential than natural selection pressure on their genomes in comparison to LSDV_2012. Moreover, relative usage in the third codon position indicates that LSDV_2019 has the highest A and T changes as compared to LSDV_ 2023 and to LSDV_2012, as shown in Figure 12D and Supplementary Data S3.

## 4. Discussion

Lumpy skin disease, caused by the LSDV, is a growing global threat to cattle, farmers and the beef industry, with significant economic impacts. The disease, marked by fever and nodules on the skin and/or organs, affects cattle of all breeds and ages, with infection rates ranging from 2% to 45% but relatively low mortality [32]. Moreover, since 2019, LSDV has spread rapidly across Asia, raising high concerns among the effected countries. Israel has also faced notable outbreaks during the 21st century in 2006, 2012–2013, 2019 and 2023. Specifically, during 2019, outbreaks in seventeen site were reported across the northern regions of Israel. Notwithstanding eradication, vaccination campaigns and control, the virus re-emerged in May 2023 in the southern part of the country. These contrasting outbreaks highlight both the virus’s persistence and the challenges of long-term control, underscoring the importance of understanding LSDV’s evolutionary dynamics and host adaptation strategies to develop effective vaccines and biosecurity measures [33,34]. In this study, we performed a comprehensive analysis of codon usage patterns, mutational pressures, non-silent mutations and APOBEC-mediated editing in the LSDV genomes from the 2019 and 2023 outbreaks. This research aims to deepen our insight into the virus’s transmission, evolution and adaptation mechanisms.

In this study, WGS was utilized to detect genetic variations in LSDV variants isolated from the 2019 and 2023 outbreaks using next-generation sequencing (NGS) and validation by Sanger sequencing followed by bioinformatics analysis.

Phylogenetic analysis was used to elucidate the evolutionary relationships among various isolates, which indicate that the LSDV_2019 and LSDV_2023 variants fall within sub-clade 1.2.1 of clade 1.2, which occupies a basal position within the genus and among ungulate viruses, sharing genetic links with sequences from South Africa and from Russia [28]. In addition, genomic gaps between the LSDV_2019 and LSDV_2023 genomes were identified, as well as single nucleotide variations. This suggests that the LSDV strains from the 2019 and 2023 outbreaks were subjected to similar evolutionary pressure that necessitated gene deletions for adaptation to the local host species or for re-emergence [35]. Moreover, deletion of nucleotides in the inter-genomic region of genes 22 and 23 of LSDV_2023 was observed when comparing it to the corresponding regions in LSDV_2019 and in LSDV_2012. Interestingly, this deletion was also found in a different LSDV sub-strain that was not known to circulate in Israel previously. Although the LSDV 2023 outbreak was detected in the southern part of the country and initial phylogenetic analysis of the EEV gene indicated a closer relationship to the outbreak reported in Egypt two years earlier (which borders Israel to the south), this deletion was not previously reported in the strains that are circulating in the region. Moreover, this deletion had only been documented a few times in LSDV strains circulating in South Asia (India in 2022 and 2024, Bhutan in 2023, Bangladesh in 2023 and 2024) and in China in 2023 [36].

In addition, we used the MEME suite, including STREME tools, for motif analysis of the inter-genomic region between genes 22 and 23 of LSDV_2019 and LSDV_2023 to identify conserved regulatory elements. This showed consistent motifs, present in both sequences, with identical positional distribution and frequency. Each motif exhibited a highly significant E-value (1.7 × 10^−1^), indicating strong enrichment and suggesting potential transcription factor binding. However, RNA secondary structure predicted that the inter-genomic region between genes 22 and 23 from LSDV_2019 produced a more stable RNA conformation in comparison to the corresponding sequence from the LSDV_2023 genome. These structural differences may influence mRNA stability, translational efficiency or interactions with RNA-binding proteins. Thus, the architecture and the divergence of the RNA structures implies that post-transcriptional regulation may exert a stronger influence on the observed biological differences than transcriptional control.

We assessed of the genetic diversity of the LSDV isolates from the 2019 and the 2023 outbreaks to evaluate gene frequencies. The analysis showed that both strains exhibited complete genome-level genetic diversity of less than 0.5% variation when compared to the LSDV_2012 genome. These findings imply that the LSDV strains from both outbreaks share a common evolutionary trajectory or pathway.

Nucleotide variations, known as single nucleotide polymorphisms (SNPs), are key contributors to genetic diversity, host–pathogen interactions and evolutionary dynamics [37]. Using MEGA X software, analysis of the nucleotide substitutions in the LSDV variants showed an increase in nucleotide substitutions, in both the LSDV_2019 and LSDV_2023 variants. However, elevated rates of transversion mutations were found in LSDV_2019 compared to LSDV_2023 at the genome level.

Additionally, the role of APOBEC enzymes, which are evolutionarily conserved and function by deaminating cytidine (C) to uracil (U) in single-stranded DNA or RNA, was examined [38]. APOBEC analysis results indicated that LSDV_2023 exhibits a higher frequency of G → A substitutions, including patterns such as GA > AA and GG > AG motif mutations, compared to LSDV_2019 and to the reference genome LSDV_2012. In particular, guanine → adenine transitions that are caused by host APOBEC enzymes play a crucial role in in the restriction of retroviruses (HIV), DNA viruses such as monkeypox virus (Mpoxv) and hepatitis B virus (HBV). Similarly, the analysis results suggest that the LSDV_2023 variant had been edited by the host’s immune system much more heavily than the LSDV_2019 variant. Thus, LSDV 2019 proposes an introduction of the virus into a new population where the hosts’ immune systems are reacting more aggressively toward it. In addition, the higher dN/dS ratio of LSDV_2019 in comparison to LSDV_2023 indicates that LSDV_2019 showed neutral evolution, whereas LSDV_2023 has negative/purifying selection. Taken together, these results suggest that APOBEC enzymes are a dominant driver in the evolution of host codon usage adaptation of LSDV.

The recombination signal detected by multiple RDP4 methods provides strong evidence that genetic exchange has occurred within the analyzed LSDV genomes. This level of concordance strongly suggests that the detected signal reflects a true recombination event rather than random similarity or alignment noise. The presence of a robust recombination event may contribute to the genetic diversity and evolutionary dynamics of circulating LSDV strains, underscoring the importance of continued genomic surveillance.

Subsequently, we used RSCU analysis as a tool to understand the evolutionary changes across different sequences or strains [39]. Using CodonW software, it was found that all 18 codon bases were utilized in all three LSDV genomes. Notably, A- and T-ending codons were predominantly used over G- and C-ending codons. However, the average RSCU in the LSDV isolates was lower than in the bovine host, suggesting low adaptation, as reported in the past in other LSDV isolates and in many other viruses as well [40].

Codon adaptation index (CAI) analysis serves as a predictor of gene expression efficiency and is instrumental in assessing codon usage bias and its relationship with host adaptation [41]. In this analysis, the CAI values for LSDV strains from LSDV_2019 and LSDV_2023 in relation to the LSDV_2012 were approximately 0.2 to 0.3, indicating a relatively low degree of adaptation to the host and less codon usage bias, which indicates neutral evolution.

Therefore, we further investigate the factors influencing codon usage bias, by applying ENc (effective number of codons), neutrality plot, and PR2 plot analyses. The PR2 plot revealed a strong preference for A and T at the third codon position in the LSDV_2019 and LSDV_2023 strains, indicating that codon usage patterns in the LSDV genomes are shaped by a complex interplay between mutational pressure and functional constraints. ENc plot analysis showed that all three LSDV genomes lie above the expected curve, reinforcing the role of mutational pressure and adaptive requirements in driving codon usage bias. Moreover, neutrality analysis demonstrated a positive correlation among the three genomes and suggested that LSDV outbreaks in 2019 and 2023 occurred due to both mutational and selection pressure. This codon bias is in agreement with earlier research reports suggesting that T/A-ended codons have a high abundance in viruses’ genomes, such as the chikungunya virus and Crimean–Congo hemorrhagic fever virus [42,43,44].

Highlighter tool employment showed that the LSDV_2023 genome sequence exhibits nucleotide mismatches, transition/transversion events and non-silent mutations at specific genomic positions in comparison to the LSDV_2019 sequence in genes 10, 11, 26, 35, 67, 88, 104, 118, 121, 122, 123, 126, 127 and 147. In addition, we have found evidence that genes 10, 11, 26, 35, 67 and 88 in LSDV_2019 had higher similarity to the LSDV_2012 isolate or the Neethling strain, which differ from LSDV_2023. However, for genes 104, 118, 121, 122, 123, 126, 127 and 147, LSDV_2023 presented higher similarity to the LSDV_2012 isolate and to the Neethling strain, while LSDV_2019 differed from them. This result suggests that recombination events between LSDV_2019 and LSDV_2012 and/or the Neethling strain during the interval years between the outbreaks that might led to the emergence of a sub-strain in the 2023 outbreak.

The three proteins with the greatest diversity, potentially affecting their activity, included protein A33, which is involved in formation of the intermediate virions; protein A34, which is involved in the intercellular and extracellular enveloped virus (EEV) production; and B4R-like ankyrin repeat protein (ANK repeat protein). Notably, the most significant non-silent mutations were observed in gene 147. Structural analysis using Chimera and AlphaFold 3 software demonstrated that the protein structures encoded by these genes in the LSDV_2019 strain differ markedly from those in the LSDV_2023 strain. Further, our analysis presented characteristics in three proteins (protein A33, A34 and ANK repeat protein) in LSDV_2019 that suggest tighter packing and stronger intra-molecular interactions in comparison to the corresponding proteins in LSDV_2023. These findings indicate that the LSDV_2023 gene products may possess weaker hydrogen bond networks and reduced conformational stability compared to their LSDV_2019 counterparts, potentially impacting protein folding, binding affinity and functional performance. Similarly, Ramachandran analysis indicates that these three proteins in the LSDV_2019 isolate adopt a different structural conformation in comparison to LSDV_2023, which may contribute to the effectiveness of the virus replication, pathogenesis and contagiousness and thus the differences in the observed outbreaks.

Despite the vaccination campaign of three years using the Neethling vaccine strain after the 2012 and 2019 outbreaks, evidence of distinct mutations originating from the LSDV_2012 or the LSDV_2019 strain can still be traced in currently circulating strains. These genomic evidences pointed that potentially recombination events between the LSDV_2012 and LSDV_2019 strains led to the emergence of a sub-strain in 2023 that had several genes with high homology to the corresponding genes in LSDV_2012, while other genes had higher homology to the corresponding genes in the LSDV_2019 strain. This dual origin of mutations might be an indication of undetectable circulation of both strains in the same region that can lead to recombination events that may give rise to new sub-strains.

Despite these insights, this study has several limitations. Due to a lack of diagnosed clinical cases between the outbreaks and no continuous genomic surveillance, our ability to reconstruct the full evolutionary pathway was limited to the major outbreak time points. Additionally, functional assays were not performed to validate the predicted structural impacts of mutations. These constraints mean that while the evidence strongly suggests mutation-driven evolution and possible recombination, the precise timing and mechanisms cannot be fully resolved.

## 5. Conclusions

The findings of this study demonstrate that LSDV has continued to evolve and persist in Israel through a combination of mutation-driven changes and possible recombination events, ultimately shaping the emergence of the 2023 variant. Although both the 2019 and 2023 isolates showed low overall genetic diversity, they accumulated distinct substitutions, inter-genic deletions, and editing signatures that indicate ongoing microevolution and potential genetic exchange with lineages not previously detected in the region. Structural differences predicted in key viral proteins further suggest that evolutionary pressures may have influenced viral stability, assembly, and host interactions, contributing to differences in outbreak behavior. Together, these patterns imply that LSDV likely circulated sub-clinically between outbreaks, enabling the gradual accumulation of mutations and facilitating recombination. However, the absence of continuous genomic surveillance restricts the ability to fully reconstruct the evolutionary pathway. Future work should prioritize routine genomic monitoring, broader sampling across regions, and functional validation of key mutations to better understand the mechanisms driving LSDV evolution and the emergence of new variants.

## Figures and Tables

**Table 1:** Complete genome levels of genetic diversity.

	LSDV_2012	LSDV_2019	LSDV_2023
**LSDV_2012**		0.00049	0.00042
**LSDV_2019**	0.003490		0.00063
**LSDV_2023**	0.002570	0.000589	

## Data Availability

The original contributions presented in this study are included in the article and Supplementary Material. Further inquiries can be directed to the corresponding author.

## Supplementary Materials

The following supporting information can be downloaded at: https://www.mdpi.com/article/10.3390/vetsci13040333/s1, Figure S1: LAST hits plot analysis of full genome deletion between isolates; Figure S2: Nucleotide mismatch, transitions and transversions, and silent and non-silent mutations analysis; Figure S3: Original image of PCR; Table S1: List of different LSDV isolates used for phylogenetic tree of EEV1 gene; Table S2: LSDV whole genomes that were used for phylogenetic analysis; Table S3: List of primers used for validation of genetic changes between LSDV_2019 and LSDV_2023 genomes; Table S4: Non-Silent mutation was analyzed through PCR in LSDV_2019 and LSDV_2023 and different genes information shown; Table S5: Values of phi/psi/Clashes/H-bond of LSDV_2019 and 2023 gene 122, 123 and 147 of different amino acids; Table S6: RSCU value of different codon of LSDV_2012, LSDV_2019 and LSDV_2023 genomes analyzed using CodonW software; Data S1: Analysis of APOBEC mutation, dN/dS ratio, and hyper mutation of LSDV_2019 and LSDV_2023 genomes with LSDV_2012; Data S2: Analysis of nucleotide/amino acid mismatch, transition/transversion, and silent/non-silent mutation in LSDV_2012, LSDV_2019, LSDV_2023 genomes; Data S3: Codon adaptation index of LSDV_2012, LSDV_2019 and LSDV_2023, analyzed using CodonW software.

## Abbreviations

The following abbreviations are used in this manuscript:

LSDV	Lumpy skin disease virus
LSD	Lumpy skin disease
NGS	Next-generation sequencing
WGS	Whole-genome sequencing
WHO	World Health Organization
BIC	Bayesian information criterion
CAI	Codon adaptation index
ENc	Effective number of codons
MAFFT	Multiple alignment fast Fourier transform
MFE	Minimum free energy
RSCU	Relative synonymous codon usage analysis
MDBK	Madin–Darby bovine kidney
DMEM	Dulbecco’s modified Eagle’s medium
FBS	Fetal bovine serum
TCID_50_	50% tissue culture infectious dose
APOBEC	Apolipoprotein B mRNA editing catalytic polypeptide-like
