# Supplementary material for: Whole-Genome Analysis of LSDV Isolates from the 2019 and 2023 Outbreaks in Israel Points to Undetected Circulation and Recombination Events
Source: Vet Sci. 2026 Mar 30;13(4):333. doi: 10.3390/vetsci13040333 (PMC13120329; doi:10.3390/vetsci13040333)
Supplement: Supplementary file 1 [file vetsci-13-00333-s001.zip › vetsci-4192306-supplementary.pdf]

Data S1: Analysis of APOBEC mutation, dN/dS ratio, and hyper mutation of LSDV_2019 and LSDV_2023 genomes with LSDV_2012.

Analysis of APOBEC between LSDV 2019 and LSDV_2023 with LSDV_2012:

**LSDV-2012**	**APOBEC**	**G→A**
**LSDV_2019**	57790	0
**LSDV_2023**	86386, 99090, 116597, 149496	26166, 93495, 120422, 130562

dN/dS ratio of LSDV_2019 and 2023 with LSDV_2012:

**Sequence names**	**Sd**	**Sn**	**S**	**N**	**ps**	**pn**	**ds**	**dn**	**ds/dn**	**ps/pn**
**LSDV_2019**	7.5	43.5	28076.5	118140.5	0.0003	0.0004	0.0003	0.0004	0.7254	0.7255
**LSDV_2023**	10.5	28.5	28935.83	121559.2	0.0004	0.0002	0.0004	0.0002	1.5479	1.5477

Hyper Mutation in LSDV_2019 and 2023 with LSDV_2012:

	**G→A**	**A→G**	**GG**	**GA**	**GC**	**GT**
**LSDV_2019**	0	1	1	0	0	0
**LSDV_2023**	4	8	2	3	2	1

<u>Data S2</u>. Analysis of nucleotide/amino acid mismatch, transition/ transversion, and silent/non-silent mutation in LSDV_2012, LSDV_2019, LSDV_2023 genomes.

Analysis of Nucleotide mismatch between LSDV 2019 and 2023 with LSDV_2012:

LSDV_2012	GAP
**LSDV_2019**	77, 78, 79, 80, 81, 82, 83, 94,85, 96, 97, 98, 99, 100, 101, 102, 103, 104, 105, 106, 107, 108, 109, 110, 111, 112, 113, 114, 115, 116, 117, 118, 119, 120, 121, 122, 123, 124, 125, 126, 127, 128, 129, 130, 131, 132, 134, 135, 136, 137, 138, 139, 140, 141, 142, 143, 144
**LSDV_2023**	13288, 15831, 15832, 15833, 15834, 15835, 15836, 15837, 15838, 15839, 15840, 15841, 15842, 15843, 15844, 15845, 15846, 15847, 99362, 135588, 137372, 140758, 140759

Analysis of Silent and Non-Silent Mutation between LSDV 2019 and 2023 with LSDV_2012:

LSDV_2012	Non-Silent Mutation
**LSDV_2019**	6175, 12037, 29641, 37987, 37996, 57172, 57790, 66889, 69097, 69106, 69109, 69112 ,69118, 69121, 69130, 69139, 76252, 81934, 84061, 92899, 93649, 99511, 99526, 99571, 107386, 107974, 111127, 111139, 111142, 111145, 113308, 114334, 116560, 123367, 140773, 140776, 148681, 148693, 148717,
**LSDV_2023**	79, 94, 2668, 6817, 7720, 18697, 28747, 29674, 40600, 44224, 68545, 86386, 91495, 101464, 113650, 116302, 116596, 116941, 120421, 121693, 122260, 122263, 122266, 122272, 130561, 144487

Analysis of Transition/transversion between LSDV 2019 and 2023 with LSDV_2012:

LSDV_2012	Transition	Transversion
**LSDV_2019**<br><br>**Transition: 8.0**<br>**Transversion: 43.0**	57790, 69147, 107387, 111143, 115849, 116560, 131766, 148718	6176, 11976, 12038, 29642, 37988, 37998, 57172, 66891, 69099, 69106, 69109, 69111, 69114, 69120, 69122, 69123, 69131, 69139, 69141, 76254, 77817, 81935, 84062, 92900, 93650, 99512, 99527, 99564, 99571, 107976, 111129, 111140, 111145, 111147, 113295, 113309, 114334, 123369, 140773, 140776, 148681, 148693, 148717
**LSDV_2023**<br><br>**Transition: 25.0**<br>**Transversion: 14.0**	79, 1687, 7721, 18698, 26166, 28023, 29675, 40600, 42288, 68545, 86386, 91495, 93495, 99090, 113652, 116597, 116942, 120422, 122260, 122273, 122274, 123363, 130562, 144487, 149496	94, 105, 2669, 6817, 28747, 44225, 101465, 116302, 121693, 122262, 122263, 122264, 122266, 122267

Data S3: Codon adaptation index of LSDV_2012, LSDV_2019 and LSDV_2023, analyzed using codon W software.

Title	T3s	C3s	A3s	G3s	CBI	GC3s	GC
**LSDV_2012**	0.4777	0.1946	0.4996	0.1758	-0.141	0.259	0.27
**LSDV_2019**	0.4825	0.1505	0.5277	0.1952	-0.177	0.235	0.268
**LSDV_2023**	0.4747	0.1673	0.5123	0.1899	-0.154	0.247	0.27

Parity plot:

	A3/A3+T3	G3/G3+C3
**LSDV_2012**	0.511	0.474
**LSDV_2019**	0.527	0.565
**LSDV_2023**	0.519	0.53

Enc-Gc Plot:

	Enc	Gc
**LSDV_2012**	47.92	0.259
**LSDV_2019**	47.31	0.235
**LSDV_2023**	47.8	0.247

Neutrality plot:

	GC3s	Gc
**LSDV_2012**	25.9	27
**LSDV_2019**	23.5	26.8
**LSDV_2023**	24.7	27.5

CAI Value:

**LSDV_2012**	0.237
**LSDV_2019**	0.228
**LSDV_2023**	0.225

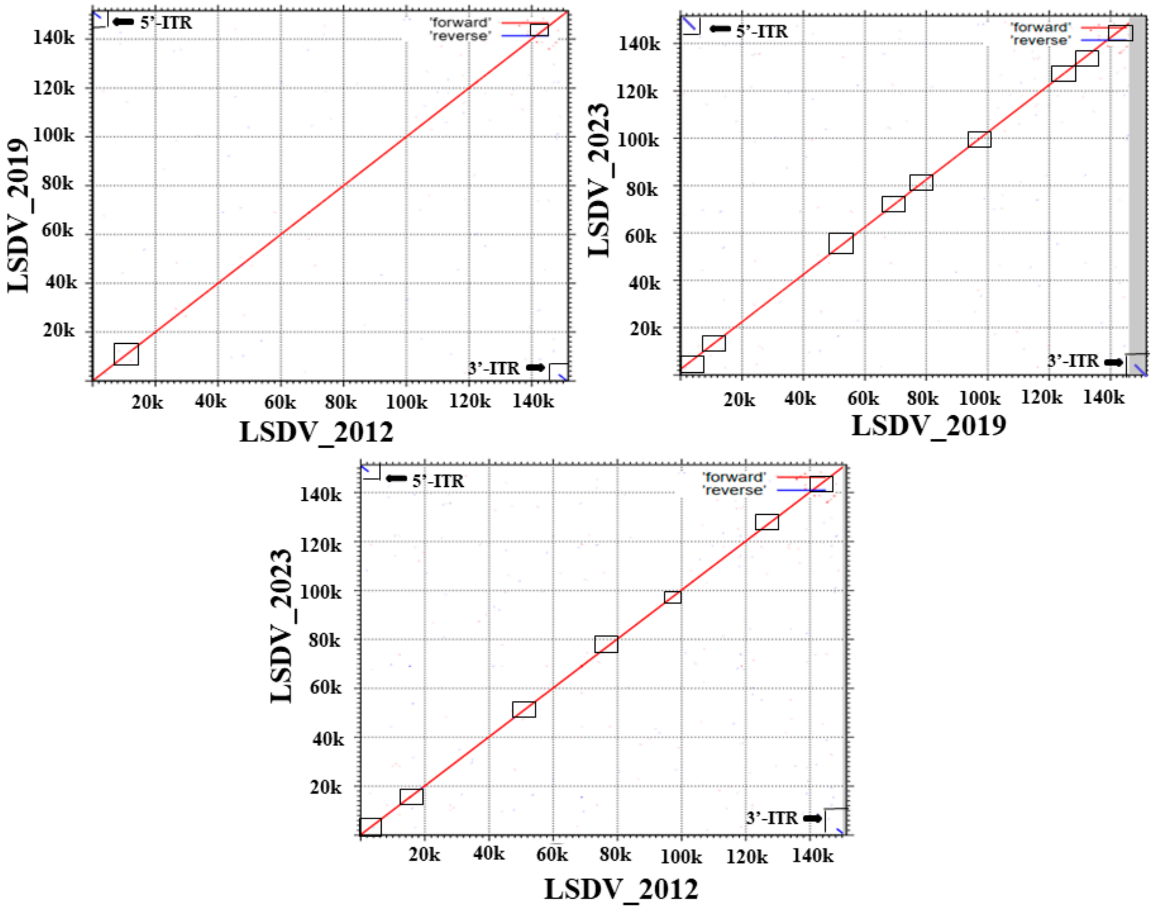

Figure S1: LAST hits plot analysis of full genome deletion between isolates.

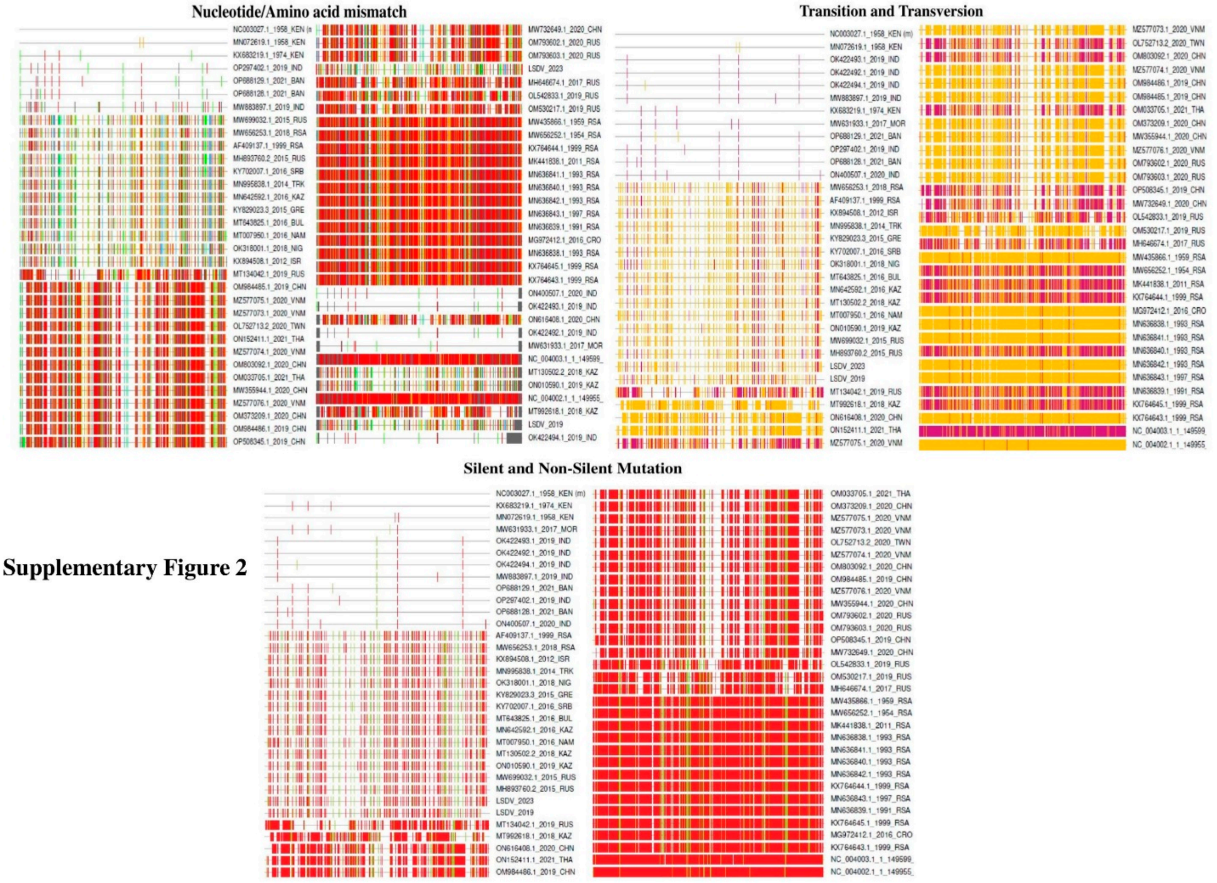

Supplementary Figure 2

Figure S2: Nucleotide mismatch, transitions and transversions, and silent and non-silent mutations analysis

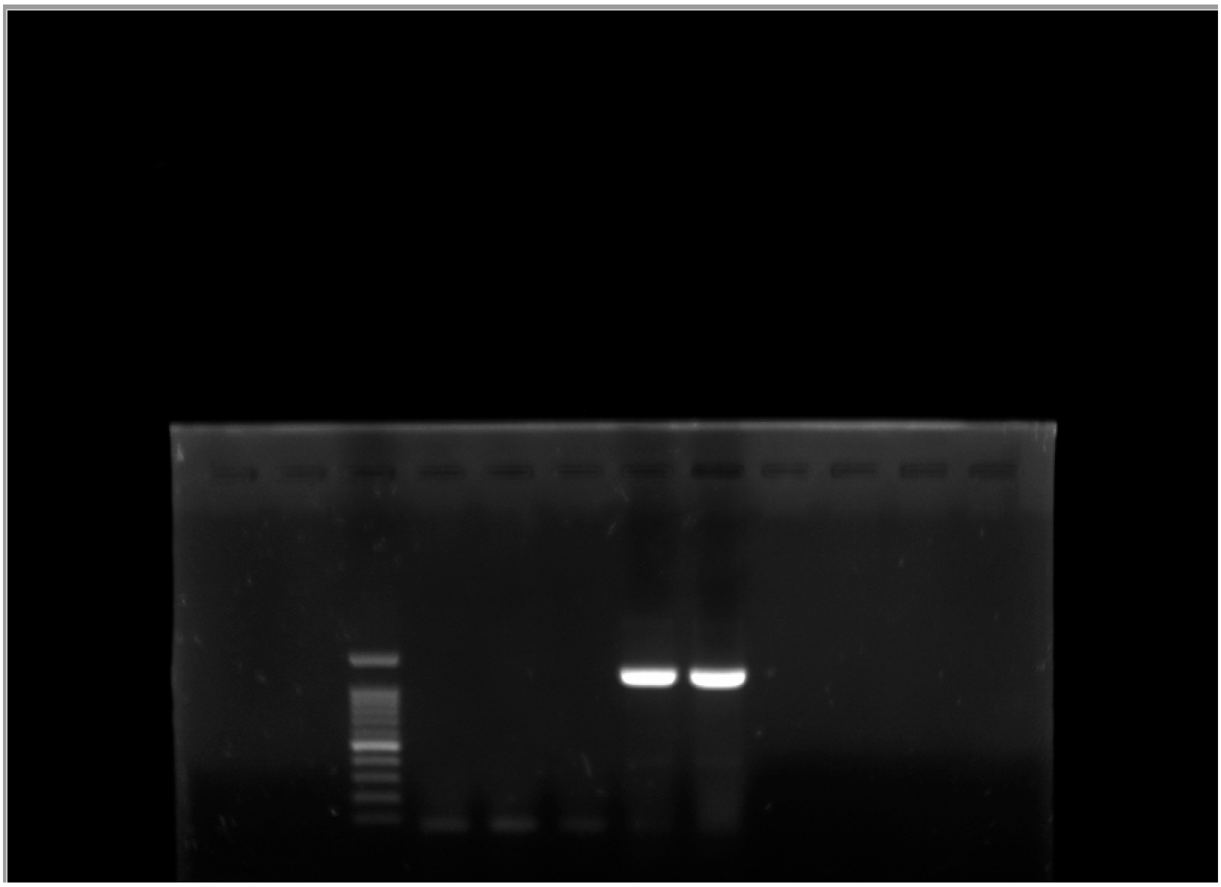

Figure S3: Original image of PCR

Table S1: List of different LSDV isolates used for phylogenetic tree of EEV1 gene

No.	GB accession	Strain	Location	year
1	MH639087	FYROM2016	Bulgaria	2016
2	MH639083	BLG99	Bulgaria	2016
3	MH639092	KSV2016	Kosovo	2016
4	MH639084	BLG172	Bulgaria	2016
5	MH639082	ALB2016	Albania	2016
6	MH639094	SNGL75	Senegal	1975
7	MW732649	LSDV/HongKong/2020	Hong Kong	2020
8	OM984485	LSDV XJ201901	China	2019
9	OP985536	LSDV/MZGD/2020/China	China	2020
10	OP752701	LSDV/FJ/CHA/2021	China	2021
11	OP654649	LSDV/China/SiC/2021	China	2021

**12**	OM105589	LSDV/China/XJ01/2019	China	2019
**13**	OM803092	China/GX01/2020	China	2020
**14**	OM803091	China/GD02/2020	China	2020
**15**	OM033705	LSDV/Thailand/YST/2021	Thailand	2021
**16**	ON616408	LSDV/NMG/2020	China	2020
**17**	OM793603	LSDV_Russia_Khabarovsk_2020	Russia	2020
**18**	OM793602	LSDV_Russia_Tomsk_2020	Russia	2020
**19**	ON152411	LSDV72/PrachuapKhiriKhan/Thailand/2021	Thailand	2021
**20**	OL752713	LSDV/KM/Taiwan/2020	Taiwan	2020
**21**	MZ577076	20L81_Bang-Thanh/VNM/20	Viet Nam	2020
**22**	MZ577075	20L70_Dinh-To/VNM/20	Viet Nam	2020
**23**	MZ577074	20L43_Ly-Quoc/VNM/20	Viet Nam	2020
**24**	MZ577073	20L42_Quyet-Thang/VNM/20	Viet Nam	2020
**25**	MW355944	China/GD01/2020	China	2020
**26**	OP508345	China/Xinjiang/Cattle/Aug-2019	China	2019
**27**	MT134042	Russia/Udmurtiya/2019	Russia	2019
**28**	OL542833	Russia/Tyumen/2019	Russia	2019
**29**	MN636843	LSD-148-GP-RSA-1997	South Africa	1997
**30**	MN636840	LSD-248-NW-RSA-1993	South Africa	1993
**31**	MN636839	LSD-103-GP-RSA-1991	South Africa	1991
**32**	MN636838	LSD-58-LP-RSA-1993	South Africa	1993
**33**	MK496636	KVI_Lib_320	Israel	2012
**34**	MK441838	Herbivac LS	South Africa	2011
**35**	MH646674	Russia/Saratov/2017	Russia	2017
**36**	MG972412	Cro2016	Croatia	2016
**37**	KX764645	Neethling-LSD vaccine-OBP	South Africa	2016
**38**	KX764644	Neethling-Herbivac vaccine	South Africa	2016
**39**	KX764643	SIS-Lumpyvax vaccine	South Africa	1999
**40**	OM793609	LSDV_Vaccine_LW-1959_1988	South Africa	1988
**41**	OM793608	LSDV_Neethling-WC_RSA_1957	South Africa	1957
**42**	OM793607	LSDV_Fourie-FS_RSA_1959	South Africa	1959
**43**	OM793606	LSDV_Potter_RSA_1958	South Africa	1958
**44**	OM793605	LSDV_Hoffmeyer_RSA_1958	South Africa	1958
**45**	OM793604	LSDV_33-KZN_RSA_1977	South Africa	1977
**46**	OM530217	Russia/Saratov/2019	Russia	2019
**47**	MW656252	Haden/RSA/1954	South Africa	1954
**48**	MW435866	SA-Neethling	South Africa	1959

**49**	MT992618	KZ-Kostanay-2018	Kazakhstan	2018
**50**	OL960034	ME-LSDV EEV126	Egypt	2021
**51**	OM373209	LSDV_BH3/CHN/20	China	2020
**52**	MH639088	ISR197	Israel	2012
**53**	ON400507	208/PVNRTVU/2020	India	2020
**54**	OK422493	Ranchi-1/P30	India	2019
**55**	OK422492	Ranchi-1/P10	India	2019
**56**	KY702007	SERBIA/Bujanovac/2016	Serbia	2016
**No.**	**GB accession**	**Strain**	**Location**	**year**
**58**	MN642592	Kubash/KAZ/16	Kazakhstan	2016
**59**	MK496638	KVI_Lib_322	Israel	2012
**60**	KX894508	155920/2012	Israel	2012
**61**	MH893760	Russia/Dagestan/2015	Russia	2015
**62**	ON010590	Neethling-RIBSP/7C	Kazakhstan	2019
**63**	ON005067	Atyrau-5BJN(IL-18)	Kazakhstan	2022
**64**	MW030512	Neethling-RIBSP(TK-)EGFP	Kazakhstan	2022
**65**	MW699032	Russia/Dagestan/2015	Russia	2015
**66**	MT130502	Neethling-RIBSP	Kazakhstan	2018
**67**	KY829023	Evros/GR/15	Greece	2015
**68**	MT643825	210LSD-249/BUL/16	Bulgaria	2016
**69**	MH639091	ISR_EZ06	Israel	2006
**70**	OK318001	V281	Nigeria	2018
**71**	MW656253	280-KZN/RSA/2018	South Africa	2018
**72**	AF409137	Neethling Warmbaths LW	South Africa	1999
**73**	MN072619	Kenya	Kenya	1958
**74**	KX683219	KSGP-0240	Kenya	1974
**75**	NC_003027	Neethling (NI-2490)	Kenya	1958
**76**	MW631933	KSGP-0240	Morocco	2017
**77**	AF325528	Neethling (NI-2490)	Kenya	1958
**78**	OP688129	V395.1	Bangladesh	2021
**79**	OP688128	V395.1	Bangladesh	2021
**80**	OP297402	WB/IND/19	India	2019
**81**	OK422494	Ranchi-1/P50	India	2019

Table S2: LSDV whole genomes that were used for phylogenetic analysis.

No.	Name	GB Accession	Clade	Year
**1.**	LSDV NI-2490, complete genome	NC003027*	1.2.2 (1.2)	1958
**2.**	LSDV isolate 155920/2012, complete genome	KX894508*	1.2.1.2 (1.2)	2012
**3.**	LSDV isolate SERBIA/Bujanovac/2016, complete	KY702007*	1.2.1.2 (1.2)	2016
**4.**	LSDV isolate Namibia_2016_9F	MT007950*	1.2.1.2 (1.2)	2016
**5.**	LSDV isolate pendik, complete genome	MN995838*	1.2.1.2 (1.2)	2014
**6.**	LSDV, complete genome	MW699032*	1.2.1.2 (1.2)	2015
**7.**	LSDV strain Kubash/KAZ/16, complete genome	MN642592*	1.2.1.2 (1.2)	2016
**8.**	LSDV strain Neethling-RIBSP/7C genomic sequence	ON010590*	1.2.1.2 (1.2)	2019
**9.**	LSDV isolate Evros/GR/15, complete genome	KY829023*	1.2.1.2 (1.2)	2015
**10.**	LSDV strain 210LSD-249/BUL/16, complete genome	MT643825*	1.2.1.2 (1.2)	2016
**11.**	LSDV isolate KZ-Kostanay-2018, partial genome	MT992618*	2.3	2018
**12.**	LSDV isolate Cro2016, complete genome	MG972412*	1.1	2016
**13.**	LSDV strain Herbivac LS batch 008, complete genome	MK441838*	1.1	2011
**14.**	LSDV isolate LSDV/Haden/RSA/1954, complete genome	MW656252*	1.1	1954
**15.**	LSDV isolate LSD-58-LP-RSA-1993, complete genome	MN636838*	1.1	1993
**16.**	LSDV isolate LSD-103-GP-RSA-1991, complete genome.	MN636839*	1.1	1991
**17.**	LSDV isolate LSD-148-GP-RSA-1997, complete genome	MN636843*	1.1	1997
**18.**	LSDV strain Neethling-Herbivac vaccine, complete genome	KX764644*	1.1	1993
**19.**	LSDV isolate SA-Neethling, complete genome	MW435866*	1.1	1959
**20.**	LSDV strain 20L43_Ly-Quoc/VNM/20, complete genome.	MZ577074*	2.5	2020
**21.**	LSDV strain LSDV/HongKong/2020, complete genome	MW732649*	2.5	2020
**22.**	LSDV isolate China/Xinjiang/Cattle/Aug-2019, complete genome	OP508345*	2.5.1 (2.5)	2019
**23.**	LSDV isolate LSDV/KM/Taiwan/2020, complete genome	OL752713*	2.5	2020
**24.**	LSDV strain LSDV/NMG/2020, partial genome	ON616408*	2.5	2020
**25.**	LSDV isolate LSDV/Thailand/YST/2021, complete genome	OM033705*	2.5.1 (2.5)	2021
**26.**	LSDV strain LSDV FJ2019, complete genome	OM984486*	2.5	2019
**27.**	isolate LSDV_Russia_Khabarovsk_2020, complete genome	OM793603*	2.5.1 (2.5)	2020
**28.**	LSDV strain Neethling-RIBSP vaccine, partial genome	MT130502*	1.2.1.2 (1.2)	2018
**29.**	LSDV strain LSDV/Russia/Saratov/2017, complete genome	MH646674*	2.1	2017
**30.**	LSDV isolate LSDV/Russia/Saratov/2019, complete genome	OM530217*	2.1	2019
**31.**	LSDV isolate Kenya, complete genome	MN072619*	1.2.2 (1.2)	1958
**32.**	LSDV isolate LSDV-WB/IND/19, complete genome	OP297402*	1.2.2 (1.2)	2019
**33.**	LSDV isolate 208/PVNRTVU/2020, complete genome	ON400507*	1.2.2 (1.2)	2020
**34.**	LSDV isolate V395.1, complete genome	OP688129*	1.2.2 (1.2)	2021
**35.**	LSDV isolate LSD, complete genome	MW631933*	1.2.2 (1.2)	2017
**36.**	LSDV strain KSGP 0240, complete genome	KX683219*	1.2.2 (1.2)	1974
**37.**	LSDV strain LSDV/Russia/Udmurtiya/2019, complete genome	MT134042*	2.2	2019
**38.**	LSDV isolate LSDV/280-KZN/RSA/2018, complete genome	MW656253*	1.2.1.1 (1.2)	2018
**39.**	LSDV isolate V281, complete genome	OK318001*	1.2.1.1 (1.2)	2018
**40.**	LSDV isolate Neethling Warmbaths LW, complete genome	AF409137*	1.2.1.1 (1.2)	1999
**41.**	LSDV isolate LSDV_BH3/CHN/20, complete genome	OM373209	2.5.1 (2.5)	2020
**42.**	LSDV isolate LSDV_Russia_Tomsk_2020, complete	OM793602	2.5.1 (2.5)	2020
**43.**	LSDV strain China/GX01/2020, complete genome	OM803092	2.5	2020
**44.**	LSDV strain LSDV XJ201901, complete genome	OM984485	2.5	2019
**45.**	LSDV72/PrachuapKhiriKhan/Thailand/2021, complete genome	ON152411	2.5	2021
**46.**	LSDV isolate LSDV/Russia/Tyumen/2019, complete	OL542833	2.4	2019
**No.**	**Name**	**GB Accession**	**Clade**	**Year**
**47.**	LSDV/Cattle/India/2019/Ranchi-1/P50, complete genome	OK422494	1.2.2 (1.2)	2019
**48.**	isolate LSDV/Cattle/India/2019/Ranchi-1/P10, complete genome	OK422492	1.2.2 (1.2)	2019
**49.**	LSDV/Cattle/India/2019/Ranchi-1/P30, complete genome	OP688128	1.2.2 (1.2)	2021

**50.**	LSDV strain LSDV/Russia/Dagestan/2015, complete genome	MH893760	1.2.1.2 (1.2)	2015
**51.**	LSDV strain 20L70_Dinh-To/VNM/20, complete genome	MZ577075	2.5	2020
**52.**	LSDV strain 20L42_Quyet-Thang/VNM/20, complete genome	MZ577073	2.5	2020
**53.**	LSDV strain SIS-Lumpyvax vaccine, complete genome	KX764643	1.1	1993
**54.**	LSDV strain Neethling-LSD vaccine-OBP, complete genome	KX764645	1.1	1993
**55.**	LSDV isolate LSD-220-1-NW-RSA-1993, complete genome	MN636841	1.1	1993
**56.**	LSDV isolate LSD-220-2-NW-RSA-1993, complete genome	MN636842	1.1	1993
**57.**	LSDV isolate LSD-248-NW-RSA-1993, complete genome	MN636840	1.1	1993
**58.**	LSDV strain 20L81_Bang-Thanh/VNM/20, complete genome	MZ577076	2.5.1 (2.5)	2020
**59.**	LSDV strain China/GD01/2020, complete genome	MW355944	2.5	2020
**60.**	isolate LSDV/Cattle/India/2019/Ranchi-1/P30, complete genome	OK422493	1.2.2 (1.2)	2019
**61.**	LSDV isolate LSDV/Cattle/India/2019/Ranchi-1	MW883897	1.2.2 (1.2)	2019
**62.**	Goatpox virus Pellor, complete genome	NC004003	-	2002
**63.**	Sheeppox virus 17077-99, complete genome	NC004002	-	2002

* LSDV genomes that were used for Simplot analysis.

Table S3. List of primers used for validation of genetic changes between LSDV_2019 and LSDV_2023 genomes.

LSDV Gene	Primers	Size	Tm
**10**	**Forward-** TGGAAGGGAGTGATAATACCAACA<br>**Reverse-** ATCACTCTTCGCGGCAAGAT	**24**<br>**20**	**59.2**<br>**59.8**
**11**	**Forward-** CCTAGCTGTAGTTCACCCAGT<br>**Reverse-** CTACTGGTGCTACGCAATCG	**21**<br>**20**	**58.8**<br>**58.8**
**26**	**Forward-**TGGTTAAATTAAAACACGGAAATGCA<br>**Reverse-** TCCCAAAGGGTTTCATCATCGT	**26**<br>**22**	**59**<br>**60**
**35**	**Forward-**ACGCGTTCTATGACAAAATCTTT<br>**Reverse-**TGTATCCTAGCTTTTTCGGAGGA	**23**<br>**23**	**57.4**<br>**59.0**
**67**	**Forward-** TGCTTGTTTCTGAAAATCTCGCA<br>**Reverse-** CCTCGTCATCGTCCGCATTA	**23**<br>**20**	**60**<br>**60**
**88**	**Forward-** TGGTATCCACCCAGATTCATCA<br>**Reverse-** TGGTATCCAAATTCAACGAAGAAGT	**22**<br>**25**	**58.6**<br>**59**
**104**	**Forward-**AGATTCCAACTTATCAACATATCCAGT<br>**Reverse-** AAGGTTTTGATAGATGGTGGTAGA	**27**<br>**24**	**58.4**<br>**57.4**
**108**	**Forward-** TCGTCAGAGACAAAGTTGCCA<br>**Reverse-** TGTCTATTTTATGCGTGTCGGT	**21**<br>**22**	**60**<br>**58.1**
**118**	**Forward-** ATGCCCGTATTCGCCACTAG<br>**Reverse-** GCTCATTCAGCGTTTGAGTT	**20**<br>**20**	**60**<br>**57**
**122**	**Forward-** TACAGCATTCGCAGGTTCCA<br>**Reverse-** AGCTTCTCATCTCGTCGCTT	**20**<br>**20**	**60**<br>**60**

**123**	**Forward-** TTGTTAGTGGTATAGGCACCG<br>**Reverse-** ACTTACGTAGTTACACGACACA	**21**<br>**22**	**58**<br>**58**
**126**	**Forward-** TCTGTTGTATACGTCGTAGTACCA<br>**Reverse-** TGACTCGGAATTATTTTGTGCA	**24**<br>**23**	**59**<br>**57**
**147**	**Forward-** ATGAATTAGCGATAAGGTCAAAGTT<br>**Reverse-** TCAGCACCGTTTCTAGTAGCA	**25**<br>**22**	**57**<br>**59.7**
**Inter-genomic region (22-23)**	**Forward-** AGCACCATCACCATCACCAT<br>**Reverse-** TGGAAAGCCATACAACAGCA	**20**<br>**20**	**59**<br>**57**

Table S4: Non-Silent mutation was analyzed through PCR in LSDV_2019 and LSDV_2023 and different genes information shown.

Gene	Amino Acid Position	LSDV_2012	LSDV_2019	LSDV_2023	Function of Gene
		Amino Acid	Amino Acid	Amino Acid	
**LSDV 10**	103	Leucine	Leucine	Phenylalanine	"LAP/PHD finger-like protein (Gene ID: 921605)
**LSDV 11**	32<br>33<br>34<br>35<br>198	-<br>-<br>-<br>-<br>Methionine	Lysine<br>Serine<br>Theronine<br>Isoleucine<br>Methionine	Lysine<br>Serine<br>Theronine<br>Isoleucine<br>Isoleucine	G protein-coupled chemokine receptor-like protein (Gene ID: 921632)
**LSDV 26**	147<br>256<br>257<br>259	Asparagine<br>Serine<br>Theronine<br>Arginine	Asparagine<br>Lysine<br>Tryosine<br>Lysine	Aspartic acid<br>Lysine<br>Tryosine Lysine	LSDV026 hypothetical protein (Gene ID: 921593)
**LSDV 35**	22<br>130<br>297	Asparagine<br>Serine<br>Valine	Aspartic acid<br>Phenylalanine<br>Valine	Aspartic acid<br>Phenylalanine<br>Alanine	LSDV 35 hypothetical protein (Gene ID: 921638)
**LSDV 67**	64<br>172	Isoleucine<br>-	Isoleucine<br>Aspartic acid	Methionine<br>Aspartic acid	putative host range protein (Gene ID: 921587)
**LSDV 88**	492	Isoleucine	Isoleucine	Arginine	Nucleoside triphosphatase I C-terminal
**LSDV 104**	01<br>04<br>16<br>21	Methionine<br>Aspartic acid<br>Glycine<br>Glycine	Isoleucine<br>Tryosine<br>Valine<br>Valine	Methionine<br>Aspartic acid<br>Glycine<br>Glycine	IMV membrane protein
**LSDV 108**	17	Alanine	Glutamic Acid	Alanine	Pox virus entry-fusion-complex G9/A16
**LSDV**	42<br>106	Theronine<br>Leucine	Alanine<br>Isoleucine	Theronine<br>Leucine Glycine	Envelope protein A28

**118**	107<br>108<br>112	Glycine<br>Phenylalanine<br>Glutamic Acid	Aspartic acid<br>Tryosine<br>Glutamine	Phenylalanine<br>Glutamic Acid	
**LSDV 122**	04<br>100<br>126<br>143<br>155<br>162<br>172<br>176<br>178<br>181	Aspartic Acid<br>Aspartic Acid<br>Glutamic Acid<br>Glutamic Acid<br>Serine<br>Tryptophan<br>Glutamic Acid<br>Glutamic Acid<br>Glutamic Acid<br>Aspartic acid	Glycine<br>Asparagine<br>Lysine<br>Lysine<br>Arginine<br>Glycine<br>Lysine<br>Lysine<br>Lysine<br>Asparagine<br>Serine	Aspartic Acid<br>Aspartic Acid<br>Glutamic Acid<br>Glutamic Acid<br>Serine<br>Tryptophan<br>Glutamic Acid<br>Glutamic Acid<br>Glutamic Acid<br>Aspartic acid	Protein A33-EEV glycoprotein
**LSDV 123**	115<br>121<br>123<br>128<br>129<br>140<br>141<br>145<br>155<br>157<br>159<br>161	Aspartic acid<br>Theronine<br>Aspartic acid<br>Theronine<br>Glutamic Acid<br>Aspartic acid<br>Isoleucine<br>Tryosine<br>Valine<br>Cysteine<br>Tryosine<br>Serine	Asparagine<br>Proline<br>Asparagine<br>Proline<br>Lysine<br>Asparagine<br>Phenylalanine<br>Phenylalanine<br>Glycine<br>Glycine<br>Asparagine<br>-	Aspartic acid<br>Theronine<br>Aspartic acid<br>Theronine<br>Glutamic Acid<br>Aspartic acid<br>Isoleucine<br>Tryosine Valine<br>Cysteine<br>Tryosine<br>Serine	Protein A34 IEV and EEV membrane glycoprotein
**LSDV 126**	85	Lysine	Glutamic Acid	Lysine	EEV glycoprotein
**LSDV 147**	03<br>04<br>07<br>10<br>14<br>15<br>17<br>19<br>20<br>21<br>23<br>24<br>32<br>34<br>40<br>43<br>48<br>49	Leucine<br>Leucine<br>Tryosine<br>Histidine<br>Valine<br>Aspartic Acid<br>Aspartic Acid<br>Valine<br>Glutamic Acid<br>Leucine<br>Isoleucine<br>Arginine<br>Glutamic Acid<br>Cystine<br>Leucine<br>Tryosine<br>Aspartic Acid<br>Valine	Arginine<br>Phenylalanine<br>Asparagine<br>Leucine<br>Glutamic Acid<br>Asparagine<br>Asparagine<br>Glutamic Acid<br>Lysine<br>Phenylalanine<br>Phenylalanine<br>Lysine<br>Lysine<br>Trytophan<br>Phenylalanine<br>Phenylalanine<br>Lysine<br>Glycine	Leucine<br>Leucine<br>Tryosine<br>Histidine<br>Valine<br>Aspartic Acid<br>Aspartic Acid<br>Valine<br>Glutamic Acid<br>Leucine<br>Isoleucine<br>Arginine<br>Glutamic Acid<br>Cystine<br>Leucine<br>Tryosine<br>Aspartic Acid<br>Valine	Ankyrin repeat domain- containing protein M-T5

	55<br>59	Arginine<br>Glutamic Acid	Lysine<br>Lysine	Arginine<br>Glutamic Acid	

"-" Deletion of Amino Acid

Table S5: Values of phi/psi/Clashes/H-bond of LSDV_2019 and 2023 gene 122, 123 and 147 of different amino acids.

**LSDV-2019-122**					**LSDV-2023-122**				
**Amino Acid**	**Phi (φ)**	**Psi (ψ)**	**Clashes**	**H bond**	**Amino Acid**	**Phi (φ)**	**Psi (ψ)**	**Clashes**	**H bond**
**Gly (4)**	**-**	**-**	**-**	**-**	**Asp (4)**	-75.0	170.6	7	2
**Asn (100)**	-53.6	-22.8	3	13	**Asp (100)**	-66.3	-32.2	4	11
**Lys (126)**	-80.3	-122.2	190	17	**Glu (126)**	-76.5	121.4	177	10
**Lys (143)**	-129.3	159.2	101	26	**Glu (143)**	-123.7	158.2	73	2
**Arg (155)**	-52.5	-31.6	1324	31	**Ser (155)**	-54.5	-28.9	2	4
**Gly (162)**					**Trp (162)**	-74.2	133.6	624	4
**Lys (172)**	-60.2	115.9	29	15	**Glu (172)**	-64.1	114.4	10	0
**Lys (176)**	-63.0	-28.1	224	16	**Glu (176)**	-57.1	109.2	6	1
**Lys (178)**	-165.6	74.1	170	4	**Glu (178)**	-64.4	93.7	32	2
**Ser (181)**	-65.7	-102.1	111	8	**Asp (181)**	-72.9	94.9	0	4
**LSDV-2019-123**					**LSDV-2023-123**				
**Amino Acid**	**Phi (φ)**	**Psi (ψ)**	**Clashes**	**H bond**	**Amino Acid**	**Phi (φ)**	**Psi (ψ)**	**Clashes**	**H bond**
**Asn (115)**	- 74.8	118.4	91	44	**Asp (115)**	-76.5	116.3	38	41
**Pro (121)**	-66.1	-141.3	4	0	**Thr (121)**	-70.9	141.9	0	1
**Asp (123)**	-77.3	104.3	9	4	**Asp (123)**	-75.4	107.6	4	5
**Pro (128)**	-55.0	-29.4	4	0	**Thr (128)**	-62.8	-37.8	1	0
**Lys (129)**	-71.3	-39.3	291	4	**Glu (129)**	-63.5	-50.4	254	23
**Asn (140)**	-87.3	117.4	34	9	**Asp (140)**	-78.3	136.9	76	12

**Phe (141)**	-92.4	143.3	116	0	**Ile (141)**	-124.9	128.7	0	0
**Phe (145)**	-124.7	130.7	275	0	**Tyr (145)**	126.9	135.9	311	10
**Gly (155)**	-	-	-	-	**Val (155)**	-141.5	136.9	5	0
**Gly (157)**	-	-	-	-	**Cyh (157)**	-63.9	-16.3	5	1
**Asn (159)**	-65.2	154.7	39	7	**Tyr (159)**	-58.1	149.8	187	0
**X (161)**	-	-	-	-	**Ser (161)**	-142.8	164.5	2	0
**LSDV-2019-147**					**LSDV-2023-147**				
**Amino Acid**	**Phi (φ)**	**Psi (ψ)**	**Clashes**	**H bond**	**Amino Acid**	**Phi (φ)**	**Psi (ψ)**	**Clashes**	**H bond**
**Arg (3)**	-52.8	-13.0	249	4	**Leu (3)**	-63.5	-32.8	33	0
**Phe (4)**	-64.5	-39.0	173	0	**Leu (4)**	-65.5	-43.0	39	0
**Asn(7)**	-85.4	-35.6	19	4	**Tyr (7)**	-63.1	-47.9	164	16
**Leu (10)**	-104.7	-18.5	13	0	**His (10)**	-107.7	-16.9	33	3
**Glu (14)**	110.5	74.4	320	42	**Val (14)**	-95.6	123.9	0	0
**Asn(15)**	-51.6	-36.2	3	12	**Asp (15)**	-97.9	139.6	27	10
**Asn (17)**	-79.9	-32.1	38	5	**Asp (17)**	-69.8	-35.2	16	1
**Lys (20)**	-63.0	-28.1	224	16	**Glu (20)**	-61.5	-43.4	300	3
**Phe (21)**	-78.1	-47.5	104	0	**Leu (21)**	-59.4	-47.7	23	0
**Phe (23)**	-70.5	-23.6	52	0	**Ile (23)**	-64.3	-44.7	23	0
**Lys (24)**	-88.8	-23.1	189	8	**Arg (24)**	-62.9	-28.3	379	16
**Asn (32)**	-80.4	-177.0	72	23	**Glu (32)**	-117.5	149.7	220	13
**Trp (34)**	-64.9	-23.2	54	1	**Cys (34)**	-63.5	17.1	0	0
**Phe (40)**	-60.9	-45.6	179	0	**Leu (40)**	-61.4	-46.3	29	0
**Phe (43)**	-59.3	-43.1	130	0	**Tyr (43)**	-60.9	-43.0	210	20
**Lys (48)**	-133.4	94.6	37	14	**Asp (48)**	-104.4	28.4	1	21
**Gly (49)**	NA	NA	NA	NA	**Val (49)**	-61.9	121.6	3	0
**Lys (50)**	-59.4	-38.2	297	14	**Arg (55)**	-59.5	-45.4	785	29

Table S6: RSCU value of different codon of LSDV_2012, LSDV_2019 and LSDV_2023 genomes analyzed using codon W software.

**AA**	**Codon**	**LSDV_2012**	**LSDV_2019**	**LSDV_2023**
Phe	UUU	1.48	1.61	1.54

	UUC	0.52	0.39	0.46
Leu	UUA	2.51	2.5	2.55
	UUG	1.11	1.07	1.11
	CUU	0.92	0.86	0.86
	CUC	0.34	0.26	0.27
	CUA	0.77	0.9	0.84
	CUG	0.35	0.41	0.37
IlE	AUU	1.21	1.13	1.17
	AUC	0.61	0.43	0.52
	AUA	1.18	1.44	1.31
Met	AUG	1	1	1
Val	GUU	1.7	1.63	1.66
	GUC	0.49	0.33	0.4
	GUA	1.26	1.4	1.32
	GUA	0.55	0.64	0.62
Ser	UCU	1.36	1.41	1.49
	UCC	0.92	0.61	0.72
	UCA	1.26	1.57	1.52
	UCG	0.49	0.56	0.54
Pro	CCU	1.12	1.04	1.12
	CCC	0.58	0.51	0.46
	CCA	1.88	1.89	1.85
	CCG	0.42	0.55	0.58
Thr	ACU	1.26	1.22	1.25
	ACC	0.67	0.48	0.51
	ACA	1.56	1.62	1.65
	ACG	0.55	0.68	0.59
Ala	GCU	1.14	1.08	1.24
	GCC	0.73	0.48	0.53
	GCA	1.58	1.78	1.62
	GCG	0.55	0.66	0.61
Tyr	UAU	1.48	1.55	1.51
	UAC	0.52	0.45	0.49
	UAA	1.72	1.78	1.71
	UAG	0.57	0.68	0.64
His	CAU	1.47	1.56	1.51
	CAC	0.53	0.44	0.49
Gln	CAA	1.53	1.46	1.46
	CAG	0.47	0.54	0.54
Asn	AAU	1.37	1.42	1.41
	AAC	0.63	0.58	0.59

Lys	AAA	1.61	1.58	1.59
	AAG	0.39	0.42	0.41
Asp	GAU	1.58	1.61	1.61
	GAC	0.42	0.39	0.39
Glu	GAA	1.57	1.58	1.56
	GAG	0.43	0.41	0.44
Cys	UGU	1.49	1.56	1.53
	UGC	0.51	0.44	0.47
	UGA	0.71	0.58	0.68
Trp	UGG	1	1	1
Arg	CGU	0.84	1.3	1.02
	CGC	0.35	0.37	0.35
	CGA	0.91	0.81	0.87
	CGG	0.32	0.42	0.39
Ser	AGU	1.44	1.46	1.28
	AGC	0.53	0.4	0.45
Arg	AGA	2.62	2.17	2.41
	AGG	0.97	0.94	0.96
Gly	GGU	1.18	1.37	1.24
	GGC	0.49	0.4	0.45
	GGA	1.74	1.62	1.57
	GGG	0.58	0.61	0.74
